# Genetic Differences in Transcript Responses to Low-Dose Ionizing Radiation Identify Tissue Functions Associated with Breast Cancer Susceptibility

**DOI:** 10.1371/journal.pone.0045394

**Published:** 2012-10-15

**Authors:** Antoine M. Snijders, Francesco Marchetti, Sandhya Bhatnagar, Nadire Duru, Ju Han, Zhi Hu, Jian-Hua Mao, Joe W. Gray, Andrew J. Wyrobek

**Affiliations:** Life Sciences Division, Lawrence Berkeley National Laboratory, Berkeley, California, United States of America; Baylor College of Medicine, United States of America

## Abstract

High dose ionizing radiation (IR) is a well-known risk factor for breast cancer but the health effects after low-dose (LD, <10 cGy) exposures remain highly uncertain. We explored a systems approach that compared LD-induced chromosome damage and transcriptional responses in strains of mice with genetic differences in their sensitivity to radiation-induced mammary cancer (BALB/c and C57BL/6) for the purpose of identifying mechanisms of mammary cancer susceptibility. Unirradiated mammary and blood tissues of these strains differed significantly in baseline expressions of DNA repair, tumor suppressor, and stress response genes. LD exposures of 7.5 cGy (weekly for 4 weeks) did not induce detectable genomic instability in either strain. However, the mammary glands of the sensitive strain but not the resistant strain showed early transcriptional responses involving: (a) diminished immune response, (b) increased cellular stress, (c) altered TGFβ-signaling, and (d) inappropriate expression of developmental genes. One month after LD exposure, the two strains showed opposing responses in transcriptional signatures linked to proliferation, senescence, and microenvironment functions. We also discovered a pre-exposure expression signature in both blood and mammary tissues that is predictive for poor survival among human cancer patients (p = 0.0001), and a post-LD-exposure signature also predictive for poor patient survival (p<0.0001). There is concordant direction of expression in the LD-exposed sensitive mouse strain, in biomarkers of human DCIS and in biomarkers of human breast tumors. Our findings support the hypothesis that genetic mechanisms that determine susceptibility to LD radiation induced mammary cancer in mice are similar to the tissue mechanisms that determine poor-survival in breast cancer patients. We observed non-linearity of the LD responses providing molecular evidence against the LNT risk model and obtained new evidence that LD responses are strongly influenced by genotype. Our findings suggest that the biological assumptions concerning the mechanisms by which LD radiation is translated into breast cancer risk should be reexamined and suggest a new strategy to identify genetic features that predispose or protect individuals from LD-induced breast cancer.

## Introduction

Human population exposures to low-dose ionizing radiation (LD, <10 cGy) are a growing medical and public health concern due to the increasing use in medical diagnostics, therapies, security screening, and exposure to emissions from nuclear power generation and unexpected events. The human breast is sensitive to radiation-induced cancer after higher doses [1] with risks depending on exposure regimen, age at exposure, and genetic background [2], [3]. However, we know remarkably little of the molecular tissue responses after LD exposures, of response mechanisms that may be protective or risky for cancer, and how individuals may vary in their tissue repair and cancer risks. The consequences of these gaps in knowledge are not trivial and there can be serious public misconceptions and fears as dramatically illustrated in Japan and the rest of the world after the radiation releases from the Fukushima reactor complex after the Great East Japan Earthquake and tsunami of 2011.

Advanced genomic technologies have demonstrated the rich molecular responses in cells and tissues exposed to LD radiation in transcriptome, metabolome, epigenome, proteome and other omics. We have learned that LD responses can vary dramatically with genetic backgrounds and that there is very little overlap between LD and HD responses at the level of genes, pathways, networks, and functions [4], [5], [6], [7]. Although the linear-no-threshold (LNT) model remains the regulatory standard for estimating LD risks [8], it is under increasing scientific challenge because of the mounting evidence that many, and maybe most, cellular and tissue responses are not linear into the LD range [7], [9]. Dose rate is also an important variable for risk, with fractionated LD and adaptive response regimens providing protection against radiation-induced cell damage, genomic damage, and cancer endpoints [10], [11], [12], [13]. In the mammary glands (MG) of mice, lifetime tumor incidence was associated with how the exposure was fractionated, ranging from full protection to additivity of risk [12]. In the mouse p53-null chimera model, 10 cGy LD exposure to the MG stroma reduced tumor latency, suggesting that LD altered the tissue microenvironment [6], although 50 cGy did not show this effect, warranting further inquiry. While LD expression studies have provided evidence for conserved as well as cell-type specific low-dose responses [4], [5], [6], [7], the roles of genetic background on resulting tissue damage and down-stream cancer risks remain poorly understood.

Mouse models facilitate exploration of the biological and genetic features that influence risk of developing MG cancer as a result of LD exposure. The risk estimates for radiation-induced breast cancer, lung cancer and leukemia do not vary significantly between humans and mice, supporting the mouse as a reasonable surrogate model [14]. We selected two inbred strains of mice that differ in their genetic susceptibility to radiation-induced MG cancer: BALB/c as more sensitive, and C57BL/6 as more resistant [14]. BALB/c mice carry two DNA-PKcs polymorphisms with reduced protein expression, reduced catalytic activity and defective non-homologous-end-joining (NHEJ) of double strand breaks [15]. But, as we will show in this report, BALB/c and C57BL/6 also vary in RNA processing and stress response functions (including other DNA repair genes) that may contribute to their genetic differences in radiation sensitivity.

Our research strategy employs a system biology approach to examine LD-induced genomic instability and expression responses (transcriptome with *in situ* protein analyses) in radiation sensitive and resistant strains, with the purpose of identifying candidate mechanisms of genetic susceptibility for LD tissue damage and cancer risks. Radiation-induced genomic instability is a hallmark of cancer, with strong evidence that it can be induced by high dose exposures. Using a sensitive flow method for detecting chromosomal damage in white blood cells, we demonstrate that high-dose exposure induces persistent genomic instability, but only in the cancer-sensitive BALB/c mice (not in C57BL/6). In contrast, LD exposure does not induce persistent genomic instability in either strain, even though BALB/c mice are more susceptible to LD-induced cancer.

**Figure 1:**
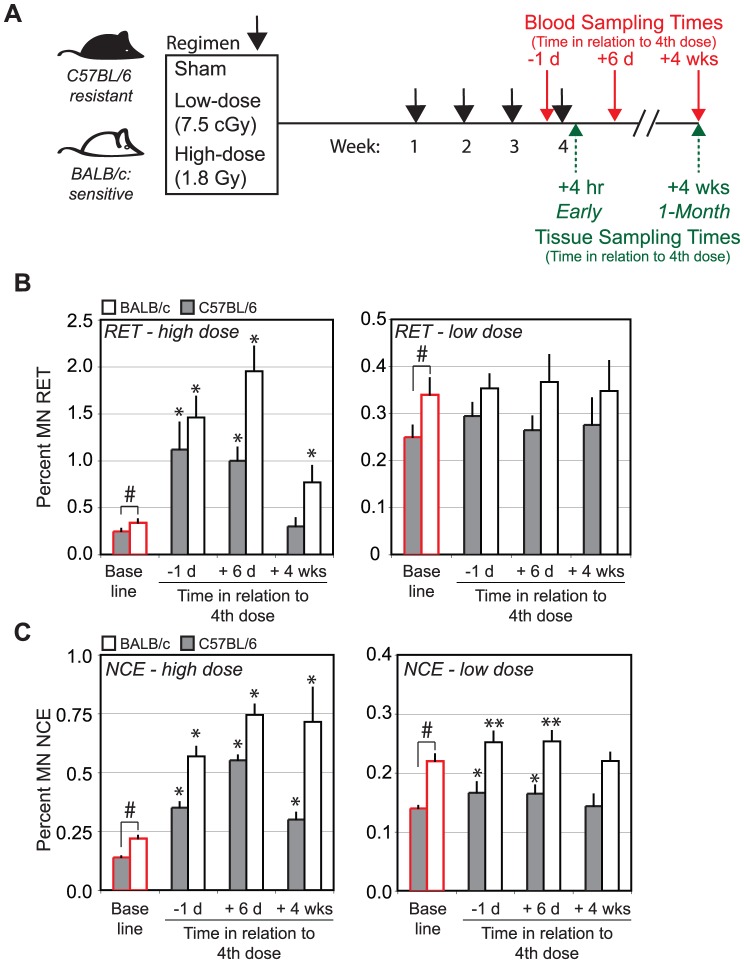
Radiation induced micronuclei in erythrocytes of mice that differ in LD-induced mammary cancer sensitivity. A. BALB/c mice are sensitive to radiation induced mammary gland, lung and ovarian cancer, whereas C57BL/6 mice are more resistant [14]. Mice were divided into three exposure groups: (1) low dose group: four weekly doses of 7.5 cGy, (2) high dose reference group: four weekly doses of 1.8 Gy and (3) unexposed group: four weekly sham irradiations (n = 6 per group). All mice were approximately 8 wks of age at the start of the radiation regimen. Saphenous vein blood was collected for micronucleus analysis at 6 days after the third dose (−1 day in relation to the 4^th^ dose), 6 days after the 4^th^ dose, and 1 month after the 4^th^ dose (n = 6 per group). Mammary gland tissues were collected for microarray (n = 4 per group) and molecular analyses at 4 hrs and 1 month after the last exposure. B. Relative frequencies of MN-RETs in high (left) and low (right) dose groups of C57BL/6 (black bars) and BALB/c mice (white bars). Bars outlined in red indicate sham-irradiated controls. C. Relative frequencies of MN-NCEs in high (left) and low (right) dose groups of C57BL/6 (black bars) and BALB/c mice (white bars). Significance was tested by ANOVA with Dunnett adjustment for multiple comparisons. An asterisk indicates significant difference in treated groups compared to the respective frequencies in sham of that same strain (* p<0.0001; ** p<0.02). The number sign (#) indicates significant baseline differences in MN RET and NCE among strains (p<0.0001).

We then launched a system search for molecular mechanisms that might explain the strain differences in breast cancer susceptibility to LD exposure using transcript profiling [5], [6], since previously we found recurrent expression changes in cell lines from unrelated individuals after doses as low as 1 cGy [7]. We investigated three exposure scenarios (Figure 1A): (1) low dose (LD) group – four weekly doses of 7.5 cGy, (2) high dose (HD) group – four weekly doses of 1.8 Gy, (3) unexposed group – four weekly sham exposures. We analyzed expression profiles to identify expression signatures associated with biological functions that might explain the differential LD cancer susceptibility between these strains. We then tested LD susceptibility associated signatures in other murine and human knowledgebases (TGFβ-responsive, pubertal mammary development, human DCIS and breast cancer biomarkers, and disease free survival in human breast cancer patients) to understand their relevance to breast cancer [16], [17], [18], [19], [20]. We test the hypothesis that genetic variation in baseline expression (i.e., expression levels before radiation exposure) and in responses to LD exposures can be used to identify tissue functions that determine susceptibility to LD-induced MG cancer and tissue functions that determine individual variation for better or poorer survival among breast cancer patients. We identified several tissue functions and two transcriptional signatures that are associated with susceptibility to LD-induced cancer in mice and with poor survival in breast cancer patients. This research lays the foundation for a new systems-biology approach for identifying the mechanisms of LD radiation-induced breast cancer, and suggests a new strategy to identify genetic features that predispose/protect individuals from risk of LD radiation-induced breast cancer.

## Results

### The baseline frequencies of micronuclei in red blood cells and transcription of 131 genes in nucleated white blood cells and mammary gland tissues differ between BALB/c and C57BL/6 female mice

We used a highly sensitive flow-cytometric assay to assess the frequency of micronucleated red cells as a measure of genome instability in unirradiated young adult female mice [21]. Figure 1B shows that the frequencies of immature reticulocytes (MN-RET) and mature normochromatic erythrocytes (MN-NCE) carrying micronuclei were ∼36% and ∼57% higher in the radiation-sensitive BALB/c strain than in the more radiation-resistant C57BL/6 strain [14] (p<0.0001; Table S1). These differences are at the high end of baseline variation among mouse strains [22], and are consistent with the significant associations that have been reported between blood micronuclei frequencies and cancer risks in human studies [23].

**Figure 2:**
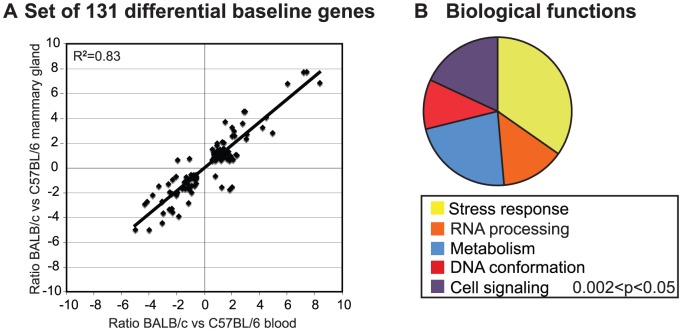
Genetic differences in baseline gene expression are correlated among tissues and associated with diverse functions. A. Transcript profiles of BALB/c (n = 4) and C57BL/6 (n = 4) mice in mammary glands and blood identified 131 genes with the same relative ratio of expression in mammary gland and blood (r^2^ = 0.83). B. Distributions of functions (GO – biological processes) associated with the 131-gene set.

We also compared the transcript profiles for nucleated cells from the peripheral blood and mammary glands (after excising the inguinal lymph nodes) from BALB/c and C57BL/6 female mice to identify common variations in gene expression and their associated tissue functions. Figure 2A shows that the BALB/c to C57BL/6 transcript ratios for the 131 genes comprising the “systemic baseline signature” are strongly correlated in blood and mammary gland cells (r^2^ = 0.83, Figure 2A). Figure 2B shows that the systemic baseline signature is significantly enriched for genes involved in stress response and RNA processing and includes DNA repair-associated genes. BALB/c tissues showed lower transcript levels than C57BL/5 for the DNA repair genes PARP3 and RAD23A (not reported previously), and higher transcript levels for MSH5 and SMC6. The PARP3, MSH5, and SMC6 expression findings were confirmed by qPCR (Table S7).

### LD did not induce or contribute to genomic instability in either strain

We measured the frequencies of micronucleated red blood cells after whole-body LD and HD exposures in sensitive BALB/c and resistant C57BL/6 female mice at the three times indicated in Figure 1A. The LD exposures induced small transient increases in MN-NCE in both strains at the two early sampling times (p<0.02), but neither strain showed evidence of radiation-induced genomic instability at 1 month after exposure (Figure 1C). The HD reference exposures (Figures 1B and 1C) increased in the frequencies of MN-RET and MN-NCE at the early sampling times in both strains (p<0.0001, Tables S2 and S3). Interestingly, the radiation-sensitive BALB/c showed significant radiation-induced genomic instability at 1 month after HD exposure while the radiation-resistant C57BL/6 mice showed no evidence of this (p<0.0001; Figure 1B, Tables S2 and S3). We confirmed this finding in a separate study of mice treated with a combined LD/HD regimen where each 7.5 cGy dose was followed 6 hr later by a 1.8 Gy dose (Tables S2 and S3). The BALB/c and C57BL/6 mice that received the combined LD/HD regimen were indistinguishable from the animals that received HD alone, confirming that the LD regimen did not induce or contribute to genomic instability in either strain.

These findings led to the hypothesis that the increased MG cancer risks in BALB/c mice after LD radiation are more likely to be associated with genetically driven differences in oncogenic barriers in their tissues. This motivated our comprehensive comparative analysis of gene expression profiles in the BALB/c and C57BL/6 strains to identify mammary tissue functions that might explain the differences in LD-induced mammary cancer rates.

### Early transcriptional responses to LD radiation are associated with immune, epithelial, and microenvironment signaling in BALB/c

**Figure 3:**
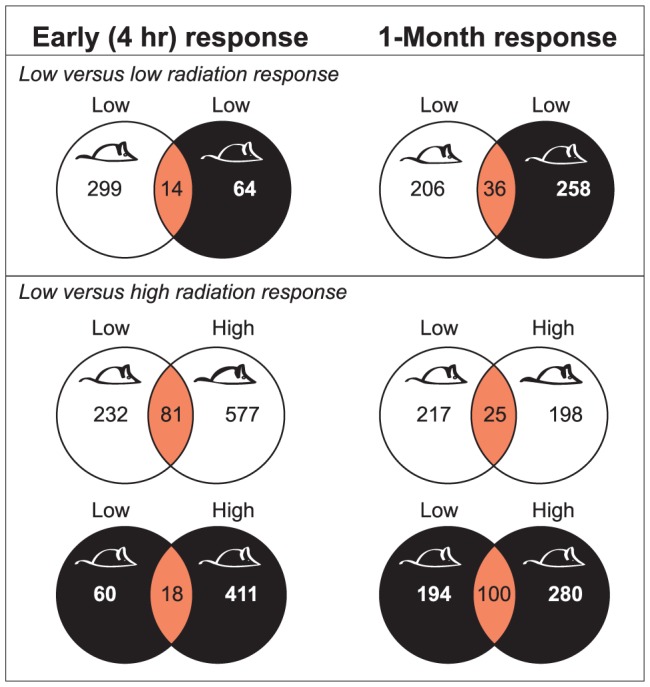
Radiation responses are dependent on genotype, dose and time after exposure. Early and 1-month response gene lists were generated based on (fold-change = 1.5 fold; p-value≤0.01 for high dose; ≤0.1 for low dose). BALB/c mice in white, C57BL/6 in black, overlap in orange.

**Figure 4:**
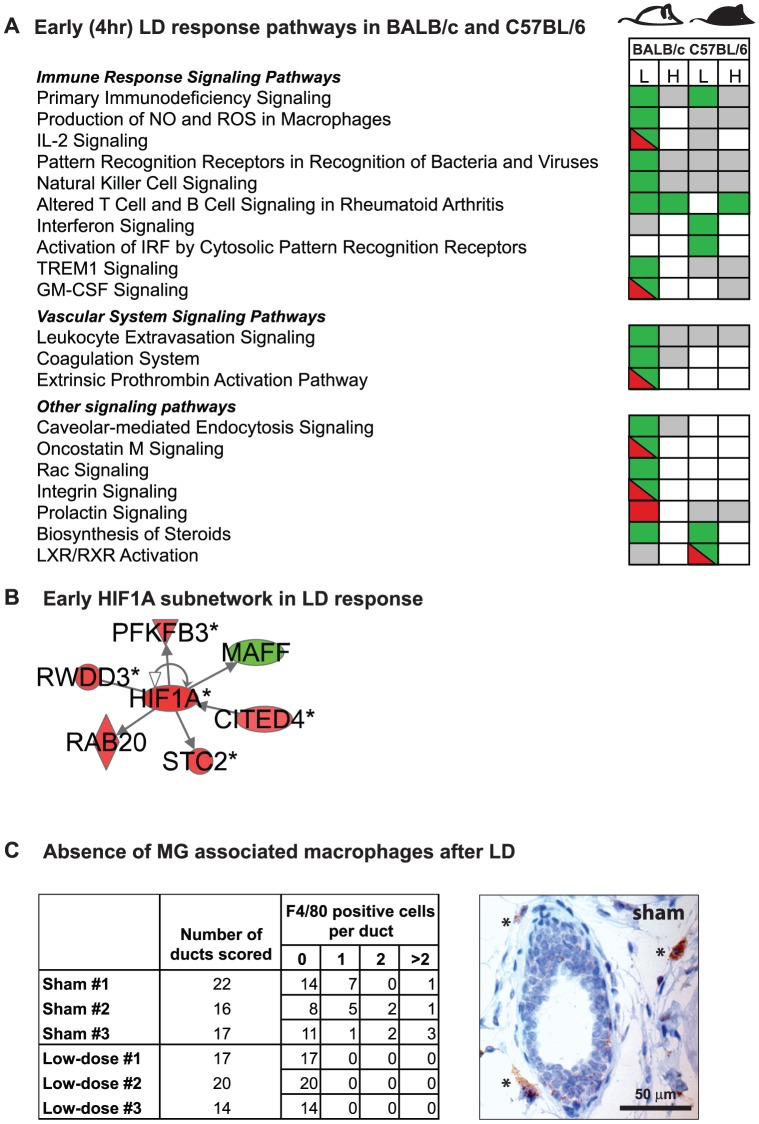
Unique early LD radiation response pathways and networks in mammary glands of BALB/c and C57BL/6. A. Early (4 hr) response for canonical pathways in BALB/c and C57BL/6 mice after low (L) or high (H) dose radiation. Downregulated pathways in green; upregulated pathways in red; pathways containing both have diagonal line; pathways not reaching statistical significance in gray. B. HIF1A Subnetwork. Protein network centered on HIF1A in the BALB/c. Downregulated genes in green; upregulated genes in red. C. Immunohistochemical (IHC) analyses of mammary glands of sham and low-dose irradiated BALB/c mice. In sham irradiated BALB/c mice at least 35% of mammary gland ducts have a surrounding macrophage. In contrast, in low-dose irradiated BALB/c mice, no macrophages were observed in 3/3 animals. A representative image of IHC analysis of EMR1 (F4/80) protein in the mammary gland of a sham irradiated BALB/c mouse is shown on the right.

Analysis of transcription profiles in MG tissues from BALB/c and C57BL/6 strains at 4 hrs (i.e., for the early response) and 1 month after LD exposure (Figure 1A; n = 4 per group) revealed response functions unique for each strain that were not induced by HD exposures. Figure 3 shows that there were ∼4× more modulated genes in BALB/c tissue than in C57BL/6 at 4 hours after LD exposure (Table S4). The differentially expressed gene sets were computationally mapped to curated functions (Figure S1A), canonical pathways (Figure 4A), and networks (Figure 4B, S2). These analyses suggested that early LD responses of BALB/c mice involved down-regulation of immune, epithelial, and microenvironment functions (14 canonical pathways, 0.002<p<0.02), which were not affected by LD in C57BL/6 nor by HD exposure in either strain (Figure 4A, and Table S5 for an inclusive listing). LD exposure in BALB/c but not C57BL/6 also altered expression in networks consistent with increased HIF1A stress response (Figure 4B), decreased immune and endothelial function (Figure 4A), and altered MG developmental- and TGFβ- regulation (Table 1).

**Table 1:** The early LD radiation response in BALB/c mice is mediated by TGFβ, involves inappropriate expression of mammary development genes, and involves breast cancer associated genes.

Gene Function	Early (4-hr) LD responsive genes in BALB/c mice (total = 313)
TGFβ responsive^a^	144 (46%)
MG development [19]	89^b^ (28%)
Breast-cancer associated [18]	41 (13%)

a TGFβ signaling and interaction database (http://actin.ucd.ie/tgfbeta/) and [20].

b 42 genes overlap with TGFβ responsive genes.

The LD induced HIF1A transcription factor network has been associated with cellular responses to genotoxic stress and low O_2_, (Figure 4B). L2L analyses (http://depts.washington.edu/l2l/) identified 11 genes upregulated in LD-exposed BALB/c mice that were also upregulated under hypoxic conditions in renal epithelial and carcinoma cell systems (4.3e-05<p<9.0e-03) [24], [25], [26]. The HIF1A, STC2 and RAB20 responses were confirmed by qPCR (Table S7).

The LD modulated immune system network in BALB/c (Figure 4A, S2; Table S5) was associated with lymphocyte activation, and expression of cytokines, chemokines, and macrophage markers. The early BALB/c responses of selected genes in this network (IRF8, IL7R and TREM2) were confirmed by qPCR (Table S7). The early BALB/c specific LD transcriptional response also was associated with down-regulation of coagulation and leukocyte extravasation signaling (Figure 4A). These functions are an essential part of the inflammatory reaction. Down-regulation of the macrophage-specific marker EMR1 (F4/80) and macrophage-associated proteins (TREM2 and GPNMB) suggested that LD-exposure led to a reduction in macrophages in BALB/c mice. This prediction was tested and confirmed by IHC in tissue sections of sham and low-dose irradiated BALB/c mice (Figure 4C; p = 0.01). There was no significant change or downregulation of the expression of any of these macrophage-associated genes in similarly treated C57BL/6 female mice.

The early BALB/c specific LD induced transcriptional response signature included∼90 MG development genes (Table 1, S6). These genes are typically expressed at 3–7 weeks of age during puberty and not at 12 weeks (the age of the irradiated mice). These genes normally are involved in terminal end-bud development (e.g. GATA3, RUNX1, MSX2 and STAT5a), differentiation and ductal branching and morphogenesis. L2L analyses also showed that the set of LD upregulated genes in BALB/c were highly associated with the developing MG of pubertal mice in other studies (p = 5.14e-30) [19]. CD24, KRT19, WNT4, AREG and IDO1 responses in BALB/c mice were confirmed by qPCR (Table S7). Importantly, ∼50% of these early BALB/c LD response genes (144/313) were TGFβ responsive (Table 1, S6; http://actin.ucd.ie/tgfbeta/, [20]). Extracellular TGFβ activation occurs in response to the generation of ROS [27] and regulates broad epithelial and stromal radiation damage response functions of the BALB/c LD genes. The differential activation of TGFβ responsive genes that were activated in BALB/c were not activated in C57BL/6 mice, indicating that there is a major genetic difference in TGFβ response to LD radiation in the MGs in these two strains. This is consistent with the increasing evidence of the regulatory role of the TGFβ response in radiation carcinogenesis of the mammary gland [6].

### Late MG transcriptional responses to LD radiation are associated with proliferation, senescence, and microenvironment function

**Figure 5:**
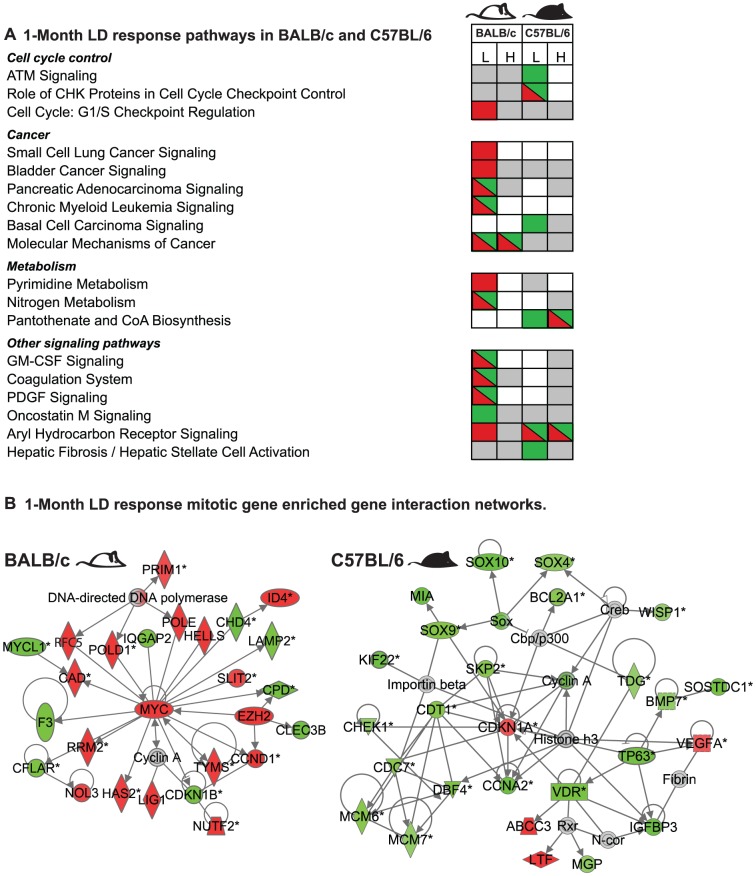
Unique 1-month LD radiation response pathways and networks in mammary glands of BALB/c and C57BL/6. A. 1-Month LD responses for canonical pathways in BALB/c and C57BL/6 mice (See legend Figure 4A). B. 1-Month LD response mitotic gene enriched interaction networks. Mitosis gene enriched interaction networks in MG of BALB/c (left) and C57BL/6 (right) mice. In the BALB/c network the majority of genes were upregulated (red) and in the C57BL/6 network the majority of genes were downregulated (green).

**Figure 6:**
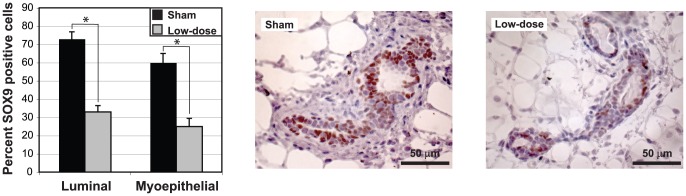
Reduction in SOX9-labelled luminal and myoepithelial cells after LD exposure in C57BL/6 mammary glands. A. The percent SOX9 positive luminal and myoepithelial cells are significantly reduced in low-dose irradiated mammary glands 1-month after low-dose exposure (p<0.0001). B. Representative images of immunohistochemical analysis of SOX9 protein in mammary glands of sham (left) or LD (right) irradiated C57BL/6 mice. Myoepithelial cells were defined as those cells directly surrounding the luminal cell layer. Counterstaining was performed using hematoxylin. Note positive staining is limited to the nucleus in cells of luminal or myoepithelial origin.

We saw a dramatic transition in the transcript profiles between the early and 1-month responses in MG tissues in radiation-sensitive BALB/c and radiation-resistant C57BL/6 strains of mice. While, similar numbers of genes were modulated in these two strains 1 month after LD exposures (Figure 3, Table S4), only a few functions significantly modulated at 4 hours in BALB/c remained so 1 month after LD exposure (Figure 5A, S1 and Table S5). One month after exposure, 5 canonical pathways were uniquely associated with C57BL/6 and a different 11 pathways were unique to BALB/c (Figure 5A). BALB/c mice acquired an enhanced proliferation phenotype (referenced to sham) while C57BL/6 acquired a diminished proliferation phenotype, consistent with senescence (Figure 5; S3). The BALB/c 1-month LD response showed upregulation of a MYC-centric network consisting of mitosis genes (Figure 5B) plus a subnetwork associated with minichromosome maintenance, (Figure S3A). In contrast, the 1-month response of C57BL/6 mice showed a highly saturated protein interaction network with the network node, CDKN1A, a negative regulator of cell cycle progression, (Figure 5B) and with down-regulation of many genes associated with DNA replication, cell-cycle progression and development. L2L analyses of the genes in the C57BL/6-specific senescence signature identified significant associations with expression signatures of cell cycle arrest and senescence (1.13E-11<p<1.15E-04; [28], [29], [30]) including downregulation of SOX9, SKP2, CCNA2 and CDKN1A (confirmed by qPCR; Table S7 and Figure 5B). SOX9 is a mark for adult human progenitor cells, mediates deposition of ECM component and is a major transcriptional regulator of mitotic activity in breast cancer [31], [32]. Consistent with its role in the control of expression of ECM, we observed down-regulation of genes associated with ECM remodeling and epithelial differentiation (not seen in BALB/c mice) suggesting a reduced turn-over of the ECM in C57BL/6 mice (Figure S3B). Figure 6 shows that expression of SOX9 protein in the MG was limited to the nuclei of luminal and myoepithelial cells and that the fraction of SOX9-positive cells was significantly reduced after LD exposure in C57BL/6 mice (p<0.0001), consistent with reduced mitotic activity in MG of C57BL/6 mice at 1 month after LD. Transcript levels of SOX9 were unaffected in similarly treated BALB/c mice.

### LD regulated genes in MG are associated with human breast cancer survival duration

We asked whether any of the strain specific baseline transcripts or LD modulated transcripts were associated with human breast cancer by using human knowledgebases that link expression profiles with breast cancer outcomes.

**Figure 7:**
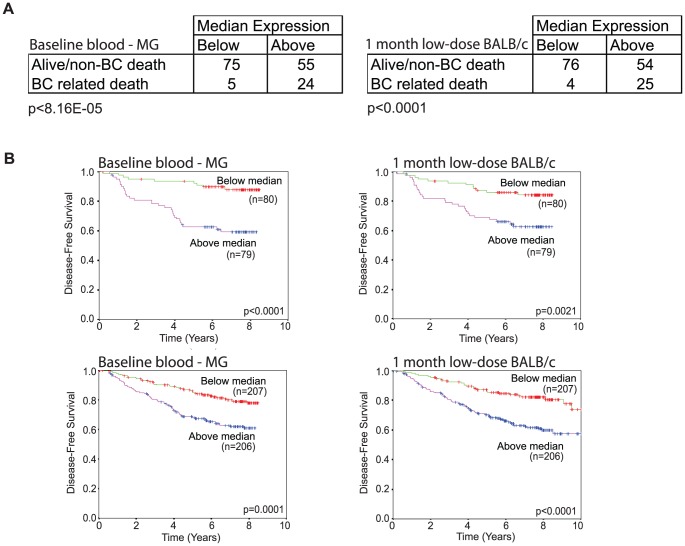
Unbiased baseline and 1-month BALB/c LD signatures are associated with human breast cancer disease-free survival. The full set of significantly differentially expressed genes between BALB/c and C57BL/6 in blood and mammary gland (left) and one-month LD up-regulated BALB/c genes (right) were used to calculate the overall sum of expression values of the same genes in human breast tumors (n = 159). A. For both signatures patients with sum expression above the group median expression had a worse prognosis than patients below the median. B. Kaplan-Meier disease-free survival curves indicate that patients with above median survival have a worse 10-year survival compared to patients with below median survival in two independent data-sets (top: GSE1456, bottom: GSE1456 and GSE6532).

We began by testing the hypothesis that expression levels of the 131 genes that are differentially expressed between non-irradiated BALB/c and C57BL6 mice in blood and MG tissues are associated with breast cancer outcomes. We tested the association of transcript levels for the 94 human orthologs that we were able to associate with the mouse baseline signature with outcome in 156 breast cancer patients for which information on disease-free survival was available [17]. We accomplished this by calculating for each patient, the sum of the normalized expression intensities of the human orthologs. As shown in Figure 7A, patients with above-median expression had significantly reduced survival duration compared to patients with below-median expression (p<8.16 E-05) and had significantly worse prognosis (Figure 7B; p<0.0001) [16], [17]. Interestingly, murine genes that showed significant strain differences only in MG or only in blood cells did not show significant associations with cancer survival, underscoring the importance of selecting genes that show “systemic differences” across tissues. As a negative control, expression levels of a set of 131 mouse genes that showed the “least” differential expression in both MG and blood between the two strains were not significantly associated with breast cancer survival (p = 0.4). Among the 94 human orthologs in the murine baseline signature, we identified 55 cancer outcome associated (COA) genes (Table 2) that individually showed differential expression between the above-median and below-median patient groups (5.3E-12<p<8.4E-03). This set of COA genes was enriched for stress response (11 genes) and RNA processing (9 genes) (Figure 2B and Table 2). Interestingly, this COA gene set contained a small number of genes (n = 9), which showed a significant association with breast cancer survival when expressed at lower levels in the above-median patient group. Among these are a number of previously proposed tumor suppressor genes: RUNX1, CBX7, PRDX2 and PRDX3. Concordant with this human finding, all four genes were also expressed at lower levels in BALB/c compared to C57BL/6 in the systemic baseline signature. This subset of 9 COA genes was significantly associated with disease free survival when down-regulated in the cancer patients (p = 5.5E-04). The signature of the remaining 46 genes was associated with disease-free survival when expressed at higher levels (55 minus 9 = 46 genes) and showed a similar association with disease-free survival as compared with the full systemic baseline signature of 94 genes (p = 6.8 E-03 vs 8.16E-05). These strain differences in baseline expression point to the importance of systemic differences in stress response, RNA processing and tumor suppressor status as candidate predictors of resistance vs susceptibility to mammary gland cancer in unirradiated individuals.

**Table 2:** Cancer Outcome Associated (COA) genes in the baseline signature are defined by their significant association with breast cancer disease-free survival.

COA Genes* of the Systemic Baseline Signature (n = 55)		
Gene	Affymetrix ID	T-test
***RNA processing genes***		
MAGOHB	218894_s_at	7.39E-05
PAPOLA	212720_at	8.98E-06
PNPT1	225291_at	5.46E-10
POP4	202868_s_at	1.99E-03
PPIH	204228_at	1.77E-05
RBM39	208720_s_at	9.41E-05
*RPS6*	209134_s_at	4.64E-03
SUPT16H	233827_s_at	7.69E-05
TXNL4A	202836_s_at	5.81E-07
***Stress response genes***		
EIF2S1	201144_s_at	1.75E-07
GNA13	224761_at	7.16E-09
GNB1	200744_s_at	8.14E-05
HLA-DRA	208894_at	2.33E-05
*PRDX2*	215067_x_at	3.39E-04
*PRDX3*	209766_at	4.39E-03
RAD23A	201039_s_at	2.33E-04
*RPS6*	209134_s_at	4.64E-03
*RUNX1*	210365_at	4.85E-06
SMC6	218781_at	2.95E-05
SUPT16H	233827_s_at	7.69E-05
***Other genes***		
ABCB10	223320_s_at	3.50E-03
ABCF1	200045_at	1.04E-09
BAT5	224756_s_at	2.01E-06
*BMP2K*	37170_at	6.77E-03
C17orf95	225808_at	5.28E-08
C19orf56	217780_at	1.64E-03
C5orf22	203738_at	5.40E-03
CAP1	213798_s_at	7.70E-06
*CBX7*	212914_at	2.68E-04
CHCHD3	217972_at	5.68E-07
CHCHD4	229595_at	1.90E-05
*CLASP2*	212308_at	8.48E-03
CLDND1	208925_at	1.06E-04
DDX19A	202578_s_at	1.52E-05
DNAJC10	221781_s_at	9.41E-12
GADD45GIP1	212891_s_at	2.13E-03
GBP1	202270_at	1.17E-05
HLA-B	208729_x_at	9.92E-06
KIF5B	224662_at	2.39E-05
MCART1	232092_at	1.86E-06
MCM6	201930_at	1.79E-07
MTFR1	203207_s_at	1.79E-07
NRD1	208709_s_at	4.64E-08
PDK1	206686_at	5.81E-06
PDXDC1	212053_at	1.30E-05
PEBP1	211941_s_at	9.61E-05
PHF20	235389_at	1.62E-03
PI4K2B	222631_at	1.87E-11
PIGO	209998_at	5.92E-03
PPME1	217841_s_at	2.47E-04
*RAB6B*	221792_at	1.08E-04
SAPS3	222467_s_at	3.76E-06
SCAND1	231059_x_at	3.21E-08
SHC1	201469_s_at	2.47E-03
*SLC15A2*	240159_at	6.86E-03
SNX6	222410_s_at	5.35E-12
TM2D2	224413_s_at	3.83E-08

* Cancer Outcome Assocaited (COA) genes were identified as follows. For each gene in the unbiased signature, a t-test was applied comparing average expression in the above median patient group vs the below median patient group. Patients were assigned to each group based on median expression of all baseline genes (94 genes). All genes with p<0.01 that were expressed at higher or lower (genes in italic) levels in the above median patient group are listed here (n = 55 genes).

We then tested the hypothesis that the 1-month BALB/c signature (i.e., the genes that are significantly upregulated at 1 month after LD exposure in relation to sham) was associated with disease-free survival among breast cancer patients. We selected the full and unbiased set of 105 BALB/c genes with significantly increased expression at 1 month after LD exposure. We examined the association of this signature with disease-free survival in breast cancer patients using two human knowledgebases that contain tumor expression profiles obtained at surgery linked to patient survival [16], [17]. Similar to our analyses of the baseline signature, we summed the expression intensities of all corresponding human orthologs (n = 96) from tumor samples and divided the patients into two groups by the median. The patients with “above median” expression values experienced higher rates of breast-cancer related deaths than “below median” patients, (Figure 7A, p<0.0001) and had significantly worse prognosis (Figure 7B; p = <0.0021) [16], [17]. As a negative control, we selected 105 of the 1-month BALB/c genes with the “least” differential expression between irradiated and sham mice, and showed that the corresponding set of human orthologs was not significantly associated with breast cancer survival (p = 0.2). Among the 96 human orthologs of the 1-month BALB/c signature, we identified 36 additional COA genes (Table 3) that individually showed differential expression between the above-median and below-median patient groups (5.4E-14<p<9.2E-03). Of these, 25 were related to mitosis, many of which have individually been associated with breast cancer survival in prior studies. Six of the 11 non-mitosis related genes were previously associated with poor survival in breast cancer patients: KRT17, MMP12, SLC7A5, SQLE, GABRP and PA2G4 [33], [34], [35], [36], [37], [38], but the remaining 5 had not been previously associated with cancer risk: CCDC86, NUP107, NUTF2, WASF1 and ELOVL6. The association between the 11 non-mitosis genes and breast-cancer-related death was comparable to using the full set of 96 human orthologs or the refined set of 36 genes suggesting strong involvement of both mitosis and non-mitosis radiation responses in defining the poor prognosis for breast cancer patients. These strain differences in 1-month post LD expression responses point to the importance of both mitosis and non-mitosis genes as candidate predictors of resistance vs susceptibility to mammary gland cancer in individuals exposed to LD ionizing radiation.

**Table 3:** Cancer Outcome Associated (COA) genes in the BALB/c 1 month LD signature are defined by their significant association with breast cancer disease-free survival.

COA Genes* of the BALB/c 1 month LD Signature (n = 36)		
Gene	Affymetrix ID	T-test
***Mitosis genes***		
CAD	202715_at	2.63E-03
CCNK	225824_at	2.07E-03
CDC7	204510_at	4.27E-06
CDT1	228868_x_at	1.21E-08
CENPH	231772_x_at	1.45E-04
CHEK1	205393_s_at	6.03E-09
EZH2	203358_s_at	8.18E-12
GINS1	206102_at	2.95E-07
HELLS	220085_at	3.39E-04
MCM2	202107_s_at	8.02E-07
MCM3	201555_at	6.17E-05
MCM4	212141_at	5.50E-07
MCM5	201755_at	4.47E-09
MCM6	201930_at	9.09E-13
MCM7	208795_s_at	2.14E-05
MYC	202431_s_at	2.92E-03
POLD1	203422_at	1.51E-04
PRIM1	205053_at	9.19E-03
RFC5	203209_at	5.46E-04
RRM2	201890_at	1.74E-13
SNRPD3	202567_at	1.18E-06
TK1	202338_at	1.14E-11
TYMS	202589_at	3.61E-09
UHRF1	225655_at	3.07E-10
WDHD1	216228_s_at	2.34E-06
***Other genes***		
CCDC86	203119_at	1.05E-05
ELOVL6	204256_at	8.42E-04
GABRP	205044_at	5.67E-06
KRT17	205157_s_at	5.87E-03
MMP12	204580_at	2.68E-05
NUP107	218768_at	5.26E-04
NUTF2	202397_at	1.34E-04
PA2G4	208676_s_at	3.67E-03
SLC7A5	201195_s_at	5.40E-14
SQLE	209218_at	1.45E-06
WASF1	204165_at	6.87E-03

* Cancer Outcome Assocaited (COA) genes were identified as follows. For each gene in the unbiased signature, a t-test was applied comparing average expression in the above median patient group vs the below median patient group. Patients were assigned to each group based on median expression of all BALB/c 1 month up genes (96 genes). All genes with p<0.01 that were expressed at higher levels in the above median patient group are listed here (n = 36 genes).

**Figure 8:**
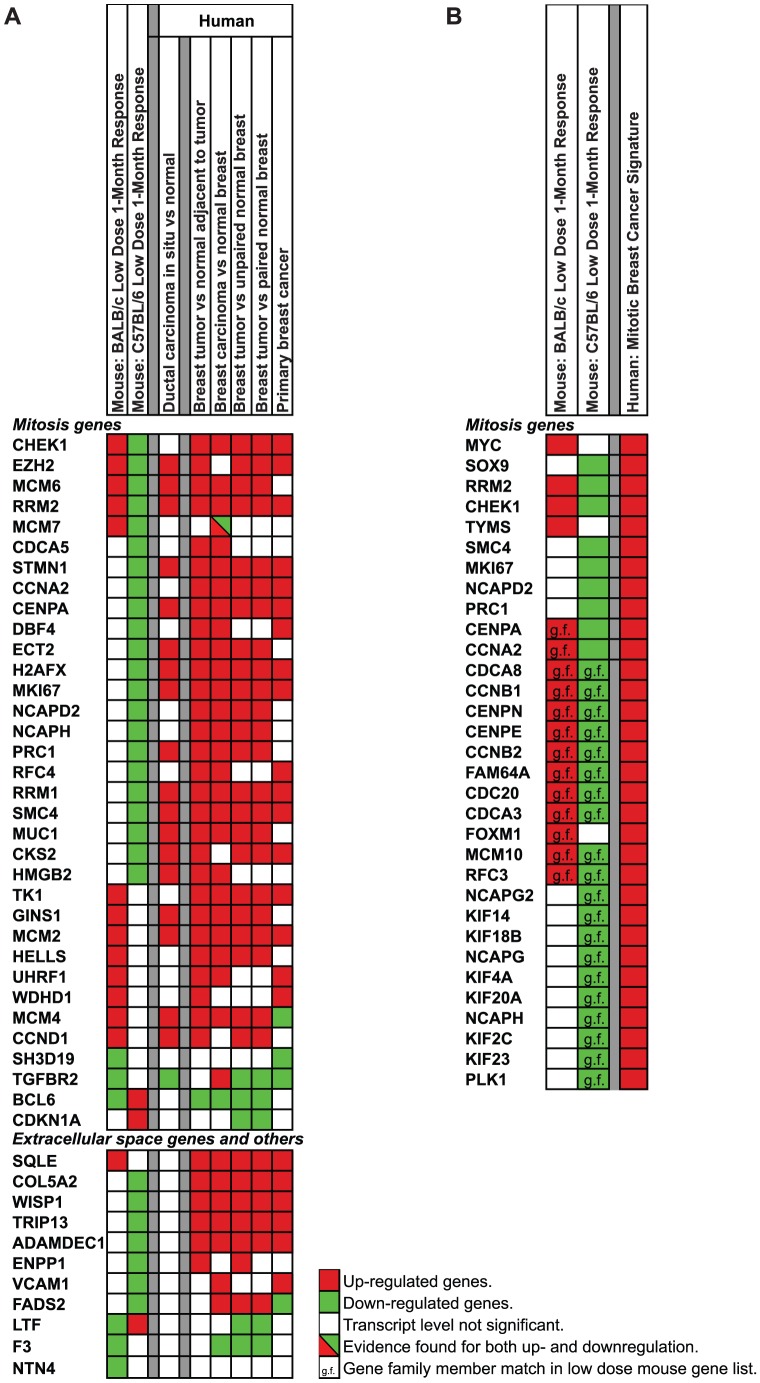
Concordance of expression between 1-month mammary gland LD radiation responses and human breast cancer signatures. A. Comparison of directionality of expression of 1-month low dose genes in BALB/c and C57BL/6 mice that overlap with 946 human breast cancer biomarkers [18] with expression in DCIS and breast cancer [39], [40], [41], [42], [43]. Upregulated genes in red; downregulated genes in green. Genes with a diagonal line had evidence for both up- and downregulation. B. A human poor prognosis signature compared against expression of 1-month low dose genes in BALB/c and C57BL/6 response genes.

We then addressed the hypothesis that the direction of expression changes in the resistant and sensitive strains at 1-month after LD exposures are concordant with the differences in gene expression of human DCIS and invasive breast cancer, and poor prognosis. (Concordance is defined as up-regulated in BALB/c, no change or down-regulated in C57BL/6 and up in human DCIS or human breast cancer; or vice versa). The BALB/c 1 month COA genes that were associated with poor prognosis when upregulated were enriched for mitosis associated genes (Table 3), whereas in C57BL/6 many of the same genes were down-regulated. As a further test of the importance of concordance in direction of expression, we compared the full set of genes modulated at 1-month after LD exposure in BALB/c and C57BL/6 mice against a meta-gene signature of 946 human breast cancer biomarkers [18], and the direction of expression for overlapping genes was compared against expression in human DCIS and breast cancer [39], [40], [41], [42], [43]. This analyses (Figure 8A) identified 45 concordant genes (34 mitosis and 11 stromal genes) with opposing responses in BALB/c and C57BL/6 where the direction of the BALB/c response matched the direction of response in independent studies of human breast cancers. Furthermore, a subset of 19 genes (Figure 8A) showed concordant responses between the mouse strains and human DCIS.

Lastly, we tested the association between the 1-month LD BALB/c signature and poor prognosis for human breast cancer. Figure 8B shows strong concordance in the direction of LD-induced expression in mammary tissue in the resistant and sensitive strains of mice and the direction of expression in patients with poor prognosis (Figure 8B). The human poor prognosis signature is under transcriptional control of SOX9, which we demonstrated to be down-regulated at both the transcript level and protein level in the resistant C57BL/6 strain (Figure 5B and 6).

## Discussion

We employed *in vivo* systems analyses to identify genetic differences in baseline (i.e., before radiation exposure) and LD-induced mammary gland gene expression in radiation-sensitive and resistant strains of mice that are at the far ends of the mammary cancer sensitivity spectrum [12], and then used *in situ* protein validation and multi-species bioinformatic resources to identify distinct tissue functions and signatures associated with cancer risks and with properties of breast cancer behavior in humans. Strain variations in baseline and LD-response expression signatures were associated with differential susceptibility to LD-induced mammary cancer in mice and with inter-individual variations in human breast cancer survival. Our findings support the hypothesis that mechanisms that control susceptibility to LD radiation induced mammary cancer in mice are similar to those that determine poor-survival in breast cancer patients. In mice, differential baseline expression of tumor suppressor genes and genes associated with stress response and RNA processing, reduced immune activity early after LD exposure and differential expression of proliferation associated genes at 1 month after LD exposure were strongly associated with higher sensitivity to LD-induced mammary tumors in mice. The strong association of baseline and 1-month signatures with disease-free survival in human breast cancer patients points to tissue mechanisms of individual variation to LD-induced mammary cancer, and provide compelling evidence for non-linear dose responses after LD exposures.

Genomic instability is a critical step in the genesis of cancers after high dose exposures [44], [45], and we like others observed that BALB/c was more sensitive to HD-induced genomic instability than C57Bl/6 [46]. Surprisingly, we found no evidence for genomic instability after LD exposures in BALB/c despite its sensitivity to LD-induced mammary cancer, which led us to search for genetic variation in molecular barrier functions that may control susceptibility to LD-induced cancer.

**Figure 9:**
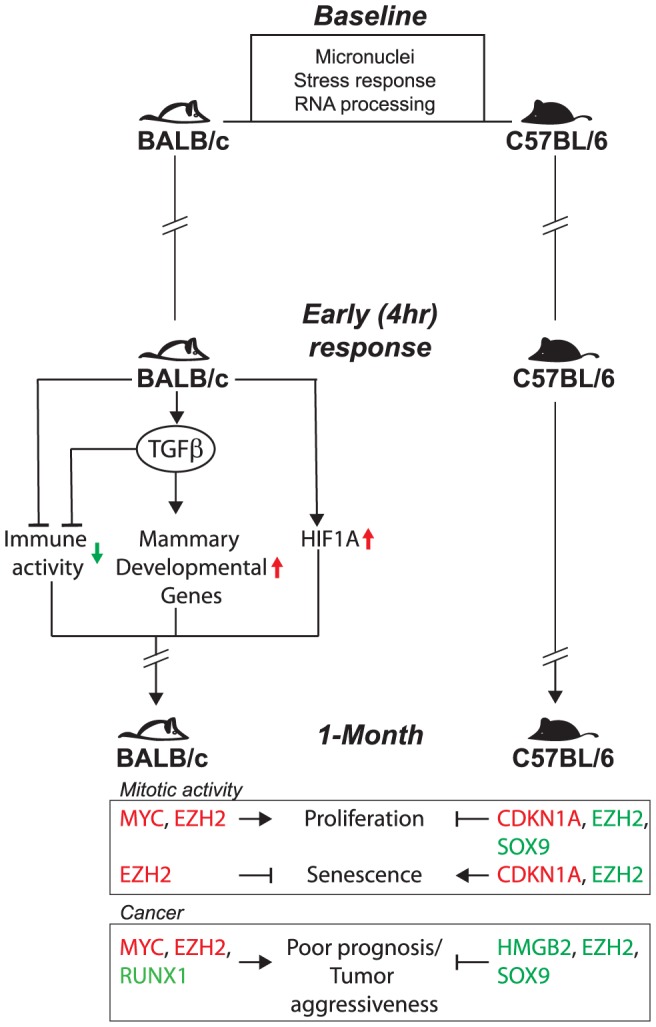
Integrative model of genetic differences in tissue functions in radiation-sensitive and resistant mice. Unirradiated BALB/c mice have significantly higher levels of micronucleated cells compared to C57BL/6. Strain differences in gene expression are associated with RNA processing and stress response and associated expression signatures are associated with poor survival in breast cancer patients. The early (4-hr) LD response in BALB/c mice is largely driven by TGFβ activation, HIF1A activity, and immune deficiency. These functions are not detected in the early expression profiles of C57BL/6 mice. At 1-month after LD irradiation, BALB/c MGs exhibits increased expression of transcriptional regulators associated with proliferation, senescence-like, and cancer-associated functions, while C57BL/6 exhibits decreased expression of proliferation-associated genes.

The genetic differences in baseline and LD expression profiles identified several unique tissue response functions associated with mammary cancer risk (Figure 9). The baseline differences in unirradiated animals (systemic signature) were significantly enriched for stress response and RNA processing genes, and included several DNA repair genes and tumor suppressor genes (Table 2). The stunted immune response observed early after LD-irradiation in BALB/c but not C57BL/6 strains was consistent with prior findings in spleen cells of LD-irradiated BALB/c mice that also received a concanavalin A challenge [47]. As predicted from the microarray data, we demonstrated in tissue sections that the stunted immune response involved reduced numbers of tissue macrophages in BALB/c mice but not C57BL/6 (Figure 4C), and there are ongoing efforts to examine differential production versus recruitment. The stunted immune response may also be related to the very high TGFβ-associated expression in BALB/c (not seen in C57BL/6 mice), given that 10 cGy exposures can activate TGFβ in BALB/c [6] which in turn can lead to immune dysfunction [48]. The early HIF1A-associated response in BALB/c may indicate tissue hypoxia or a more generalized tissue stress response [49]. Among the 12 hypoxia-inducible genes in irradiated BALB/c [24], [25], [26], both HIF1α and STC2, a HIF1A target gene, were overexpressed in human cancers and pre-neoplastic breast lesions [50]. The early expression of the transcriptional activators and repressors (i.e., STAT5a, GATA3, RUNX1 and MSX2) may be the reason for the inappropriate expression of the “pubertal-like” mammary development genes in BALB/c mice, which in their TGFβ-modified microenvironment may be associated with the increased cancer risk observed in that strain. In studies with human keratinocytes, the promoter regions of genes modulated by LD radiation were enriched for GATA3 binding sites, which supports the hypothesis that GATA3 plays an important role in the LD transcriptional response in BALB/c but not C57BL/6 [51].

Finally, our model predicts that at 1-month after LD exposures, the MG tissue of the resistant C57BL/6 mice appears to mount a barrier against cell cycle progression and ECM remodeling, similar to a senescence-like phenotype, which may block the division of residual damaged cells, thereby acting like a global tumor repressor [52]. In support of this prediction, we demonstrated that both the transcript levels of SOX9 in mammary tissue and the frequencies of SOX9-protein-positive epithelial cells were reduced in mammary tissue of C57BL/6 (but not in BALB/c) at 1 month after LD exposures. In contrast, similarly treated BALB/c mice did not show any significant changes in SOX9 transcripts. We used fold-change and graded p-value criteria to generate our initial LD gene lists to provide sufficient numbers of genes for downstream bioinformatics analyses, which is not meant to address the biological meaningfulness of individual genes. Rather it is the beginning of a process to identify candidate tissue functions and pathways that require follow-on validation, such as: (a) confirm tissue expression of genes associated with candidate functions and pathways using molecular and biochemical methods (ie, IHC for *in situ* protein expression) and (b) testing the prediction of candidate genes and functions in independent animal or human gene expression profiling data sets. We applied both of these confirmatory approaches in our paper.

Our transcript and protein expression analyses measured the individual responses of four individuals per experimental group. Indeed, we expected young adult females to be cycling through estrous. However, given the nature of our study design (multiple timed fractionated exposures and rigid sampling times), we did not synchronize animals for estrous cycle at radiation nor at sampling. Instead, we relied on the group response to “average” out the possible effects of estrous cycling. Future studies are underway to investigate whether estrous cycling affects baseline expression and LD responses of mammary tissue. Our findings also warrant additional studies using traditional genetic linkage analyses in mice as well as molecular manipulation of the expression of candidate genes to determine whether the baseline and LD response functions that we have discovered are determinants for breast cancer susceptibility.

Comparative systems analyses of the expression profiles in unirradiated mice (i.e., baseline signature) and human breast cancer outcomes identified 55 genes, each significantly associated with patient survival. In the majority of genes, poor survival was associated with increased expression. Unexpectedly, 9 genes showed the inverse association, including the tumor suppressor genes: RUNX1, CBX7, PRDX2 and PRDX3. These genes were expressed at lower levels both in the blood and MG tissues of unirradiated BALB/c compared to C57BL/6 suggesting that increased cancer sensitivity could be associated with less effective tumor suppressor mechanisms in BALB/c. CBX7 is a known tumor suppressor in both mice and humans and several PRDXs have been shown to have tumor preventive functions [53], [54], [55]. RUNX1 was special in our study, in that it was further down-regulated in BALB/c at 1 month after LD exposure. RUNX1 is a classic tumor suppressor gene in acute myeloid leukemia (AML) and loss of RUNX1 causes hyperproliferation and abnormal morphogenesis in a 3D model of breast epithelial cells [56], [57]. The differential baseline expression of tumor suppressor genes, multiple DNA repair and stress response genes in normal blood and mammary tissue of unirradiated BALB/c mice raises the intriguing hypothesis that the collective influence of the systemic functions that we have discovered, which are not themselves directly associated with mitotic status, predispose BALB/c mice to mammary cancer.

Comparative systems analyses of expression profiles at 1-month after LD exposure in mice and human breast cancer outcomes identified 36 genes that were each associated with patient disease free survival in cancer patients when they were upregulated. This signature includes mitosis-associated genes (Table 3), consistent with the observation that human cancer signatures include proliferation genes and that increased proliferation status of tumors is strongly associated with poor survival [58]. It is noteworthy that the statistical association between the 11 non-mitosis genes and breast-cancer-related death was comparable to using all 36 genes suggesting strong involvement of both mitosis and non-mitosis radiation responses in the 36 gene signature. Five of the 11 non-mitosis genes had not been previously associated with breast cancer survival. We observed minimal overlap between our signatures and the ‘intrinsic’ gene signature that defines the molecular breast cancer subtypes (SQLE and KRT17) and the 70-gene poor prognosis signature (MCM6) [59], [60], suggesting that our LD radiation-response signatures are different from those developed independently of radiation exposures and provide new information related to breast cancer risks from LD radiation exposures. These findings support the hypothesis that certain mechanisms that control susceptibility to low-dose radiation induced mammary cancer in mice are similar to those that determine poor-survival in breast cancer patients.

Our studies provide strong evidence for dose non-linearities in expression and tissue functions after LD exposures, and strong evidence against the validity of the LNT for molecular responses. The LNT model uses linear extrapolations of cancer risk from high to LD exposures, with the assumption that underlying mechanisms are also linear. Our study provides overwhelming evidence for dose non-linearities in gene expression (Figure 3), tissue functions (Figure S1) and canonical pathways (Figure 4; Table S5). We also found a number of genes showing plateau-like responses with dose. As shown in Figure S4, 76 BALB/c genes were modulated in the same direction and at similar magnitudes after low- versus high-dose exposures, in striking contrast to the 24-fold difference in doses. We also found opposing directions for low- versus high-dose responses (Figure S4B). But most surprising in regards to non-linearity was our finding of strain differences in low-dose thresholds of induction, with the sensitive BALB/c strain showing lower thresholds. Table 4 lists genes similarly modulated at high dose in both BALB/c and C57BL/6 but that differed dramatically in their low-dose responses. While the BALB/c low dose responses were generally lower compared to their high-dose responses, none of these genes were induced after low dose in C57BL/6. Interestingly, the magnitude of the C57BL/6 high-dose responses were significantly different from the BALB/c high-dose responses (p = 0.003), but were not different from the BALB/c low-dose responses (p = 0.7). Taken together our findings provide strong evidence that the high dose response is not an enhancement of the low-dose response, rather it is remarkably different and strongly argues, at least at the gene expression level, against using the LNT model for low-dose risk predictions.

**Table 4:** Strain differences in the induction of LD ‘thresholded’ genes as evidence for genetic differences in LD response mechanisms.

Gene	Low^1 ^BALB/c	High^1 ^BALB/c	Low^2 ^C57Bl/6	C57Bl/6 High^1^
Hist1h2ad	−1.23	−1.84	**Ns**	−1.25
Mpeg1	−1.18	−1.13	**Ns**	−1.13
Fbn2	−1.11	−1.66	**Ns**	−1.08
H19	−1.01	−1.56	**Ns**	−1.68
Cdt1	−0.94	−1.14	**Ns**	−0.99
Clec7a	−0.92	−1.41	**Ns**	−1.19
Mcm5	−0.89	−1.15	**Ns**	−0.60
Irf8	−0.87	−1.06	**Ns**	−0.71
Cdt1	−0.85	−1.11	**ns**	−0.75
Stmn1	−0.84	−1.48	**ns**	−1.11
Lst1	−0.81	−1.12	**ns**	−0.58
Uhrf1	−0.71	−1.13	**ns**	−0.84
Col9a1	−0.71	−1.82	**ns**	−0.89
Irf8	−0.70	−1.53	**ns**	−1.07
Fyb	−0.69	−1.04	**ns**	−1.02
Tlr1	−0.69	−0.72	**ns**	−0.60
Mcm6	−0.69	−1.02	**ns**	−1.18
Gzma	−0.68	−1.08	**ns**	−0.89
Cybb	−0.66	−0.98	**ns**	−0.66
Ptprc	−0.64	−1.21	**ns**	−1.00
Ccl5	−0.61	−0.79	**ns**	−0.91
Copg2as2	0.65	0.85	**ns**	0.82
Fbxo21	0.66	0.62	**ns**	1.08
Anxa8	0.69	1.04	**ns**	0.63
Zbtb16	0.70	1.13	**ns**	0.90
Zbtb16	0.76	1.13	**ns**	0.74
Cdh13	0.96	0.84	**ns**	0.63
Zbtb16	1.19	1.46	**ns**	1.10

1 Fold change (log_2_) with respect to sham irradiated animals (p<8.7E-02).

2 ns - not significantly modulated after low dose with respect to sham.

The fractionated low-dose exposure regimen used in our study is relevant to various human LD radiation exposure scenarios. The maximum yearly allowable dose for radiation workers in the recent nuclear crisis at a Japanese nuclear power plant is 100–250 mSv, which is similar to the whole body fractionated dosing used in our study (4×75 = 300 mSv). Also, multiple abdominal CT scans can yield doses of ∼60 mGy and full body CT scans can involve doses of ∼100 mGy, similar to the individual doses in our study. Also, the penumbra of radiotherapy fields for breast cancer can deliver doses to the contralateral breast similar to the doses in our study [2].

On the assumption that there is substantial genetic variation in molecular tissue responses and mammary cancer risks in human women exposed to LD ionizing radiation, as observed in mice, our findings provide a novel approach for developing predictive tools to identify individual with higher or lower cancer risks from LD exposures, and for distinguishing breast cancers induced by LD radiation versus other causes. Our work also points to a re-examination of the assumptions associated with biological processes controlling transduction of low-dose radiation into breast cancer risk and suggest a new strategy to identify genetic and molecular features that predispose or protect individuals from LD-induced breast cancer.

## Materials and Methods

### Ethics Statement

Female, virgin C57BL/6 and BALB/c mice (∼6 weeks old; Harlan Laboratories, Livermore, CA) were acclimatized for 2 weeks, and the study was carried out in strict accordance with the Guide for the Care and Use of Laboratory Animals of the National Institutes of Health. The protocol was approved by the Committee on the Ethics of Animal Experiments of the Lawrence Berkeley National Laboratory (Approval number: 25001). At 8 weeks of age, mice (n = 6 per group) were exposed to 4 weekly doses of (a) 7.5 cGy, (b) 1.8 Gy, or (c) sham, using a Pantak 320 kVp X-ray machine, operated at 300 kV (2 mA and 196 mGy/min for low dose, 10 mA and 783 mGy/min for high dose).

### Micronucleus Analyses

For the analyses of micronucleated red blood cells, peripheral blood was collected from each mouse (n = 6 per group) at 6 days after each weekly irradiation and at 28 days after the fourth irradiation. Approximately 100 µl of blood was collected per time point from the saphenous vein [61] and processed with the MicroFlow^BASIC^ kit for the mouse (Litron Laboratories, Rochester, NY) according to the manufacturer's instructions. Samples were kept at −80°C until shipment to Litron Laboratories where they were analyzed by flow cytometry for the frequencies of micronucleated reticulocytes (MN-RET) and micronucleated normochromatic erythrocytes (MN-NCE) [21]. Frequencies of MN-RET and MN-NCE of exposed mice were compared against the respective frequencies in sham of that same strain by ANOVA with Dunnett adjustment for multiple comparisons. A p-value less than 0.05 was considered significant. Differences in baseline MN frequencies were compared using two-tailed T-test with unequal variance.

### RNA isolation, expression profiling and bioinformatics

At 4 hours and 1-month after the last exposure we harvested the 4^th^ pair of mammary glands and removed their inguinal lymph nodes; mice were randomized and individually processed for RNA isolations (See Text S1, for details). Microarray hybridizations were carried out on each of four independent mice per dose group (n = 4) using Affymetrix's HT Mouse Genome 430A 96-Array Plate. The data has been deposited at NCBI GEO with accession number GSE40066 (http://www.ncbi.nlm.nih.gov/geo/). RMA was used to create an expression matrix and NUSE was used to assess array quality. The following bioinformatics software tools and databases were used (see Text S1, for details.): L2L (http://depts.washington.edu/l2l/), KEGG (http://bioinfo.vanderbilt.edu/webgestalt/) DAVID (http://david.abcc.ncifcrf.gov/), pubertal mammary gland development genes [19], TGFβ-responsive genes (http://actin.ucd.ie/tgfbeta/ and [20]), 942 biomarkers of breast cancer [18] and gene expression in human DCIS and breast cancers (http://www.nextbio.com/).

### Human breast cancer datasets of disease-free survival

Expression levels of human orthologs of overlapping genes in blood and mammary gland tissue of unirradiated BALB/c and C57BL/6 mice and an unbiased set of 105 up-regulated 1-month BALB/c-specific low dose genes (i.e., genes not up-regulated in C57BL/6) were summed in breast cancer samples of patients from two independent curated breast cancer data sets (GSE1456 and GSE6532) [16], [17]. The median expression value was used as a cut-point to assess group survival outcomes. A Kaplan-Meier disease-free survival curve was generated for patients with above median and below median expression. Log-rank tests were performed to compare the difference in disease-free survival between patients in the two clusters. See Text S1 for details.

## Supporting Information

Figure S1 **Tissue functions associated with the early and 1-month LD response in BALB/c and C57BL/6 mammary glands.** Distributions of predicted functions (based on GO, KEGG, IPA Canonical Pathways, Genes of Interest) at early (4 hr) and 1-month sampling times in both strains. Numbers of genes used to generate charts are listed (unique genes in parentheses).
(EPS)

Figure S2 **Early LD radiation response networks in the mammary glands of BALB/c mice show broad downregulation of immune response genes.** The top two protein interaction networks for the BALB/c low dose genes include the functions of Inflammatory Disease/Cell Mediated Immune Response/Cellular Movement (top) and Cellular Growth, Proliferation/Hematological System Development, and Function/Humoral Immune Response (bottom). Upregulated and downregulated genes are represented in red and green, respectively.
(EPS)

Figure S3 **Genetic differences in protein-interaction networks in mammary glands of BALB/c and C57BL/6 mice at 1-month after LD exposures.** A. Gene interaction network enriched for genes involved in DNA replication containing mostly MCM family genes were found to contain mostly upregulated genes (highlighted in red) in mammary gland of BALB/c mice at 1 month after low dose radiation exposure. B. Protein interaction analyses of the mammary gland of C57BL/6 mice at 1-month after low dose radiation show an extracellular-matrix network and a keratin-enriched network. Genes upregulated after low dose radiation are shown in red and downregulated genes in green. In each of these networks, the majority of genes were downregulated.
(EPS)

Figure S4 **Early expression non-linearities in the mammary glands of BALB/c mice exposed to LD radiation.** A. Fold changes in responses were calculated with respect to sham-irradiated mammary glands for genes modulated after low dose and high dose radiation exposures, represented in blue and orange, respectively. Genes were filtered on fold change (+/−1.5) and p-value (<0.1 low dose and <0.05 high dose). 76 BALB/c genes were modulated in the same direction and at similar magnitudes after low- versus high-dose exposures (left two panels). These genes are enriched for DNA metabolism (p = 0.002), DNA replication (p = 0.009) and immune responses (p<0.05) as well as mitosis. Note the large cluster of 35 genes that was differentially modulated, i.e., upregulated after low dose and downregulated after high dose (far right panel). B. Mammary epithelial markers with opposite direction of response after low and high dose exposures in BALB/c. The markers that are associated with mammary epithelium are upregulated after low dose radiation, whereas after high dose radiation the same genes were found to be downregulated.
(EPS)

Table S1 **Baseline levels of micronucleated reticulocytes (RET) and normochromatic erythrocytes (NCE) are significantly higher in BALB/c compared to C57BL/6 mice.**
(PDF)

Table S2 **Time-course of micronuclei (MN) induction in blood reticulocytes (RET) and normochromatic erythrocytes (NCE) of C57BL/6 female mice after fractionated exposures to low and high doses of ionizing radiation.**
(PDF)

Table S3 **Time-course of micronucleus (MN) induction in blood reticulocytes (RET) and normochromatic erythrocytes (NCE) of BALB/c female mice after fractionated exposures to low and high doses of ionizing radiation.**
(PDF)

Table S4 **Number of genes modulated after low and high dose exposures in the mammary glands of BALB/c and C57BL/6 at early (top) and late (bottom) timepoints after exposure.**
(PDF)

Table S5 **Canonical pathways significantly modulated after fractionated low or high dose exposures in the mammary gland of BALB/c or C57Bl/6.**
(PDF)

Table S6 **BALB/c early response genes associated with TGFβ response, MG development, and breast cancer.**
(PDF)

Table S7 **Summary of quantitative RT PCR confirmations of microarray findings.**
(PDF)

Text S1 **Supplementary methods.**
(PDF)
