# Supplementary material for: Genetic Differences in Transcript Responses to Low-Dose Ionizing Radiation Identify Tissue Functions Associated with Breast Cancer Susceptibility
Source: PLoS One. 2012 Oct 15;7(10):e45394. doi: 10.1371/journal.pone.0045394 (PMC3471924; doi:10.1371/journal.pone.0045394)
Supplement: Table S1 — Baseline levels of micronucleated reticulocytes (RET) and normochromatic erythrocytes (NCE) are significantly higher in BALB/c compared to C57BL/6 mice. (PDF) [file pone.0045394.s005.pdf]

Table S1. Baseline levels of micronucleated reticulocytes (RET) and normochromatic erythrocytes (NCE) are significantly higher in BALB/c compared to C57BL/6 mice.

	**Time[b]**	**C57BL/6**	**BALB/c**	**p-value**
**Micronucleated RET[a]**	-1 day	0.27 ± 0.03	0.33 ± 0.05	
	6 days	0.22 ± 0.02	0.34 ± 0.05	
	28 days	0.25 ± 0.05	0.37 ± 0.08	
	average	0.25	0.34	<0.0001
**Micronucleated NCE[a]**	-1 day	0.14 ± 0.01	0.23 ± 0.02	
	6 days	0.14 ± 0.01	0.23 ± 0.02	
	28 days	0.13 ± 0.00	0.20 ± 0.01	
	average	0.14	0.22	<0.0001

[a]Percent ± standard deviation
[b]Time in relation to 4th sham
