# Supplementary material for: Genetic Differences in Transcript Responses to Low-Dose Ionizing Radiation Identify Tissue Functions Associated with Breast Cancer Susceptibility
Source: PLoS One. 2012 Oct 15;7(10):e45394. doi: 10.1371/journal.pone.0045394 (PMC3471924; doi:10.1371/journal.pone.0045394)
Supplement: Table S2 — Time-course of micronuclei (MN) induction in blood reticulocytes (RET) and normochromatic erythrocytes (NCE) of C57BL/6 female mice after fractionated exposures to low and high doses of ionizing radiation. (PDF) [file pone.0045394.s006.pdf]

Table S2. Time-course of micronucleus (MN) induction in blood reticulocytes (RET) and normochromatic erythrocytes (NCE) of C57BL/6 female mice after multiple exposures to low and high doses of ionizing radiation.

**Dose (cGy)**[a]	**Time (days)**[b]	**Mice n**	**Total RET**	**MN-RET (% ± S.D.)**		**Total NCE**	**MN-NCE (% ± S.D.)**
sham (0 Gy)		6[c]	359,113	887 (0.25 ± 0.02)		15,076,138	20,390 (0.14 ± 0.004)
3 x 7.5	-1	6	119,649	351 (0.30 ± 0.03)[f]		4,525,395	7,519 (0.17 ± 0.02)[d]
3 x 180	-1	6	118,656	1,344 (1.12 ± 0.28)[d]		2,273,975	7,983 (0.35 ± 0.02)[d]
3 x (7.5 + 180)	-1	6	118,685	1,315 (1.10 ± 0.28)[d]		1,656,437	6,015 (0.36 ± 0.04)[d]
4 x 7.5	6	6	119,684	316 (0.27 ± 0.03)		5,444,232	9,097 (0.17 ± 0.01)[d]
4 x 180	6	6	118,801	1,199 (1.00 ± 0.14)[d]		573,214	3,160 (0.55 ± 0.02)[d,e]
4 x (7.5 + 180)	6	6	118,824	1,176 (0.98 ± 0.22)[d]		793,657	4,773 (0.60 ± 0.07)[d,e]
4 x 7.5	28	5	99,725	275 (0.28 ± 0.06)		4,719,142	6,896 (0.14 ± 0.02)
4 x 180	28	6	119,643	357 (0.30 ± 0.09)		3,535,137	10,692 (0.30 ± 0.03)[d]
4 x (7.5 + 180)	28	6	119,605	395 (0.33 ± 0.08)		3,064,364	9,638 (0.32 ± 0.05)[d]

[a]Once a week with 6 hr of separation for multiple daily doses
[b]In relation to 4th irradiation
[c]For each mouse, an overall average was obtained by pooling the values from the tree collection points
[d]P<0.0001 vs sham
[e]P<0.0001 vs 3 weeks
[f]Borderline significance p=0.05 vs sham
