# Supplementary material for: Genetic Differences in Transcript Responses to Low-Dose Ionizing Radiation Identify Tissue Functions Associated with Breast Cancer Susceptibility
Source: PLoS One. 2012 Oct 15;7(10):e45394. doi: 10.1371/journal.pone.0045394 (PMC3471924; doi:10.1371/journal.pone.0045394)
Supplement: Table S3 — Time-course of micronucleus (MN) induction in blood reticulocytes (RET) and normochromatic erythrocytes (NCE) of BALB/c female mice after fractionated exposures to low and high doses of ionizing radiation. (PDF) [file pone.0045394.s007.pdf]

Table S3. Time-course of micronucleus (MN) induction in blood reticulocytes (RET) and normochromatic erythrocytes (NCE) of BALB/c female mice after multiple exposures to low and high doses of ionizing radiation.

**Dose (cGy)[a]**	**Time (days)[b]**	**Mice n**	**Total RET**	**MN-RET (% ± S.D.)**		**Total NCE**	**MN-NCE (% ± S.D.)**
sham (0 Gy)		6[c]	318,926	1,074 (0.34 ± 0.04)		14,582,445	32,750 (0.22 ± 0.01)
3 x 7.5	-1	6	119,578	422 (0.35 ± 0.03)		5,242,933	11,578 (0.25 ± 0.02)[f]
3 x 180	-1	6	118,251	1,749 (1.46 ± 0.22)[d]		2,113,742	11,933 (0.57 ± 0.04)[d]
3 x (7.5 + 180)	-1	6	118,006	1,994 (1.67 ± 0.12)[d]		3,211,504	18,131 (0.56 ± 0.04)[d]
4 x 7.5	6	6	119,563	437 (0.37 ± 0.06)		4,708,513	11,959 (0.25 ± 0.02)[f]
4 x 180	6	6	117,662	2,338 (1.95 ± 0.26)[d]		1,064,685	7,945 (0.75 ± 0.04)[d,e]
4 x (7.5 + 180)	6	6	117,887	2,113 (1.76 ± 0.17)[d]		1,246,772	10,076 (0.81 ± 0.07)[d,e]
4 x 7.5	28	4	79,725	275 (0.35 ± 0.06)		4,719,142	6,896 (0.22 ± 0.01)
4 x 180	28	6	119,080	920 (0.77 ± 0.18)[d]		3,902,522	28,089 (0.72 ± 0.15)[d]
4 x (7.5 + 180)	28	6	119,057	943 (0.79 ± 0.13)[d]		3,159,295	22,067 (0.71 ± 0.09)[d]

[a]Once a week with 6 hr of separation for multiple daily doses.
[b]In relation to 4th irradiation.
[c]For each mouse, an overall average was obtained by pooling the values from the tree collection points
[d]P<0.0001 vs sham
[e]P<0.0001 vs 3 weeks
[f]P<0.02 vs sham
