# Supplementary material for: Genetic Differences in Transcript Responses to Low-Dose Ionizing Radiation Identify Tissue Functions Associated with Breast Cancer Susceptibility
Source: PLoS One. 2012 Oct 15;7(10):e45394. doi: 10.1371/journal.pone.0045394 (PMC3471924; doi:10.1371/journal.pone.0045394)
Supplement: Table S4 — Number of genes modulated after low and high dose exposures in the mammary glands of BALB/c and C57BL/6 at early (top) and late (bottom) timepoints after exposure. (PDF) [file pone.0045394.s008.pdf]

Table S4. Number of genes modulated after low and high dose exposures in the mammary glands of BALB/c and C57BL/6 at early (top) and late (bottom) timepoints after exposure.

Early response

Strain	Dose	Regimen	Criteria for gene selection		Unique genes	Up-	Down-
			Fold change	p-value		regulated	regulated
C57BL/6	4 x 7.5 cGy	Low	>0.58	<0.1	78	34	44
BALB/c	4 x 7.5 cGy	Low	>0.58	<0.1	313	146	167
C57BL/6	4 x 1.8 Gy	High	>0.58	<0.01	429	115	314
BALB/c	4 x 1.8 Gy	High	>0.58	<0.01	658	190	468

1-Month response

Strain	Dose	Regimen	Criteria for gene selection		Unique genes	Up-	Down-
			Fold change	p-value		regulated	regulated
C57BL/6	4 x 7.5 cGy	Low	>0.58	<0.1	294	75	219
BALB/c	4 x 7.5 cGy	Low	>0.58	<0.1	242	103	139
C57BL/6	4 x 1.8 Gy	High	>0.58	<0.01	380	166	214
BALB/c	4 x 1.8 Gy	High	>0.58	<0.01	223	56	167
