# Supplementary material for: Genetic Differences in Transcript Responses to Low-Dose Ionizing Radiation Identify Tissue Functions Associated with Breast Cancer Susceptibility
Source: PLoS One. 2012 Oct 15;7(10):e45394. doi: 10.1371/journal.pone.0045394 (PMC3471924; doi:10.1371/journal.pone.0045394)
Supplement: Table S5 — Canonical pathways significantly modulated after fractionated low or high dose exposures in the mammary gland of BALB/c or C57Bl/6. (PDF) [file pone.0045394.s009.pdf]

Table S5. Canonical pathways significantly modulated after fractionated low or high dose exposures in the mammary gland of BALB/c or C57Bl/6.

EARLY (4 hr) RESPONSE	BALB/c		C57BL/6	
	L	H	L	H
p53 Signaling				
Cell Cycle: G2/M DNA Damage Checkpoint Regulation				
Cdc42 Signaling				
Cell Cycle: G1/S Checkpoint Regulation				
Primary Immunodeficiency Signaling				
Production of Nitric Oxide and Reactive Oxygen Species in Macrophages				
TREM1 Signaling				
Leukocyte Extravasation Signaling				
IL-2 Signaling				
Role of Pattern Recognition Receptors in Recognition of Bacteria and Viruses				
Natural Killer Cell Signaling				
GM-CSF Signaling				
Altered T Cell and B Cell Signaling in Rheumatoid Arthritis				
B Cell Development				
Calcium-induced T Lymphocyte Apoptosis				
CTLA4 Signaling in Cytotoxic T Lymphocytes				
Cytotoxic T Lymphocyte-mediated Apoptosis of Target Cells				
Allograft Rejection Signaling				
Autoimmune Thyroid Disease Signaling				
Graft-versus-Host Disease Signaling				
Nur77 Signaling in T Lymphocytes				
Antiproliferative Role of TOB in T Cell Signaling				
OX40 Signaling Pathway				
IL-4 Signaling				
Antigen Presentation Pathway				
Interferon Signaling				
Activation of IRF by Cytosolic Pattern Recognition Receptors				
Chemokine Signaling				
LPS-stimulated MAPK Signaling				
CCR5 Signaling in Macrophages				
T Helper Cell Differentiation				
Toll-like Receptor Signaling				
B Cell Receptor Signaling				
CD28 Signaling in T Helper Cells				
Biosynthesis of Steroids				
Glutathione Metabolism				
Methionine Metabolism				
Nicotinate and Nicotinamide Metabolism				
Prolactin Signaling				
Coagulation System				
Caveolar-mediated Endocytosis Signaling				
Oncostatin M Signaling				
Rac Signaling				
Extrinsic Prothrombin Activation Pathway				
Integrin Signaling				

Hepatic Fibrosis / Hepatic Stellate Cell Activation
DNA Methylation and Transcriptional Repression Signaling
HER-2 Signaling in Breast Cancer
Aryl Hydrocarbon Receptor Signaling
VDR/RXR Activation
Glioblastoma Multiforme Signaling
LXR/RXR Activation
Role of Lipids/Lipid Rafts in the Pathogenesis of Influenza
Thrombopoietin Signaling
Docosahexaenoic Acid (DHA) Signaling
Molecular Mechanisms of Cancer
Wnt/b-catenin Signaling
14-3-3-mediated Signaling
RAR Activation
HGF Signaling
Type I Diabetes Mellitus Signaling
Glioma Signaling

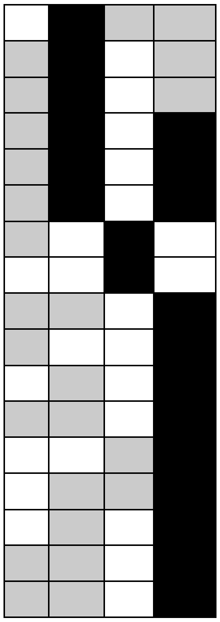

1-MONTH RESPONSE

BALB/c		C57BL/6	
L	H	L	H

ATM Signaling
Role of CHK Proteins in Cell Cycle Checkpoint Control
Pyrimidine Metabolism
Cell Cycle: G1/S Checkpoint Regulation
p53 Signaling

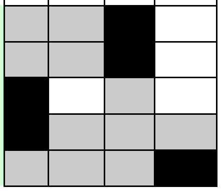

IL-12 Signaling and Production in Macrophages
LPS-stimulated MAPK Signaling
IL-22 Signaling
IL-3 Signaling
LPS/IL-1 Mediated Inhibition of RXR Function
GM-CSF Signaling
TNFR1 Signaling

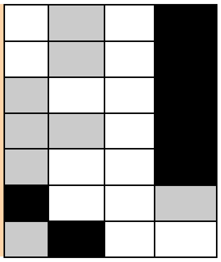

Nitrogen Metabolism
Pantothenate and CoA Biosynthesis
Glycolysis/Gluconeogenesis
Ascorbate and Aldarate Metabolism
Riboflavin Metabolism
Starch and Sucrose Metabolism
Glycerolipid Metabolism
Glycerophospholipid Metabolism
Nicotinate and Nicotinamide Metabolism
Metabolism of Xenobiotics by Cytochrome P450

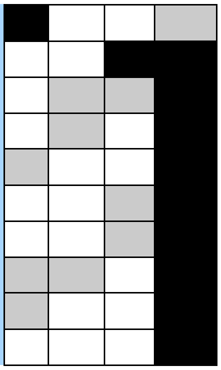

PDGF Signaling
Coagulation System
Oncostatin M Signaling
Small Cell Lung Cancer Signaling
Pancreatic Adenocarcinoma Signaling
Chronic Myeloid Leukemia Signaling
Bladder Cancer Signaling

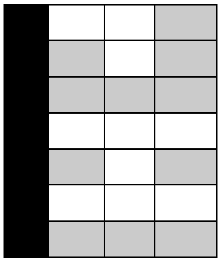

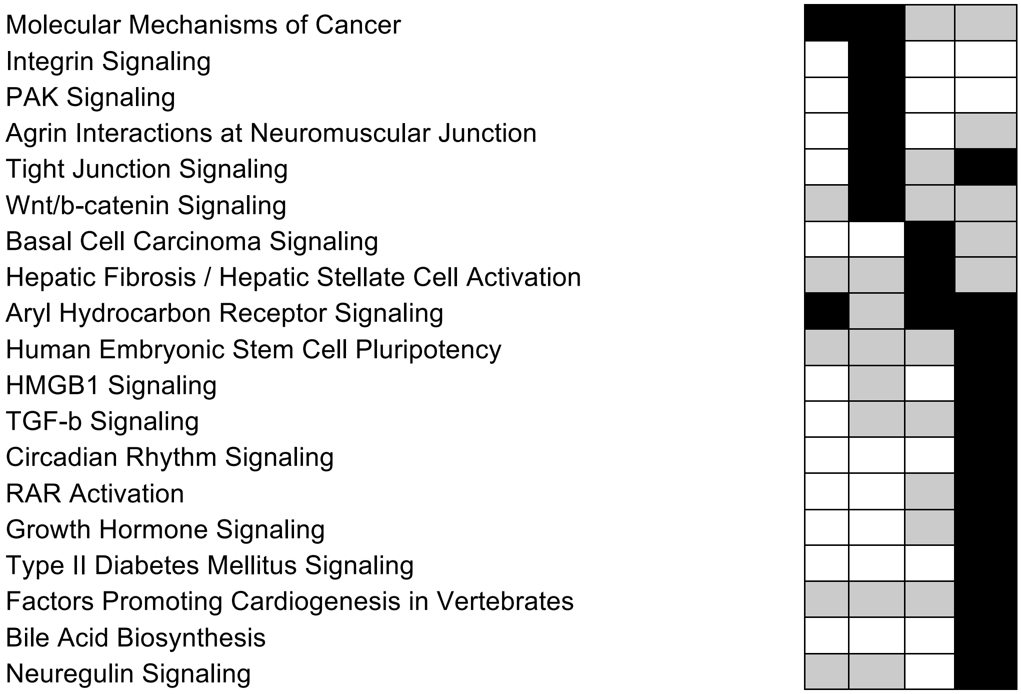
Molecular Mechanisms of Cancer
Integrin Signaling
PAK Signaling
Agrin Interactions at Neuromuscular Junction
Tight Junction Signaling
Wnt/b-catenin Signaling
Basal Cell Carcinoma Signaling
Hepatic Fibrosis / Hepatic Stellate Cell Activation
Aryl Hydrocarbon Receptor Signaling
Human Embryonic Stem Cell Pluripotency
HMGB1 Signaling
TGF-b Signaling
Circadian Rhythm Signaling
RAR Activation
Growth Hormone Signaling
Type II Diabetes Mellitus Signaling
Factors Promoting Cardiogenesis in Vertebrates
Bile Acid Biosynthesis
Neuregulin Signaling
