# Supplementary material for: Genetic Differences in Transcript Responses to Low-Dose Ionizing Radiation Identify Tissue Functions Associated with Breast Cancer Susceptibility
Source: PLoS One. 2012 Oct 15;7(10):e45394. doi: 10.1371/journal.pone.0045394 (PMC3471924; doi:10.1371/journal.pone.0045394)
Supplement: Table S6 — BALB/c early response genes associated with TGFβ response, MG development, and breast cancer. (PDF) [file pone.0045394.s010.pdf]

Table S6. BALB/c early response genes associated with TGFβ response, MG development, and breast cancer*.

**A.** ***TGFβ-responsive*** [a] ***(n=144)***				**B.** ***MG Development*** [b] ***(n=89).***		**C.** ***Breast cancer-associated*** [c] ***(n=41).***
Abp1	Ptpro	Tmem30b	S100a8	Adam10	Mafb	Gata3
Adam10	Rgs1	Tspan1	Sdc3	Akr1C14	Malat1	Hspa1B
Anxa8	Slc40a1	Usp2	Spp1	Anxa8	Mboat1	Tspan1
Arg1	Smarcc1	Zbtb16	Stmn1	Aqp5	Mbp	Pde4Dip
Atrx	Syngr1	Abcg1	Syk	Areg	Mcm6	Mbp
Cradd	Top2a	Ada	Tnfaip6	Arg2	Mmd2	Ptger3
F3	Abca1	Adam19	Tnrc18	Atp1A3	Mpz	Clca2
Fxyd2	Asns	Atf5	Tubb2a	Ccnd2	Prlr	F3
Gadd45g	Cd24a	Bach1	Xrn1	Cd24	Runx1	Ltf
Gpx3	Cdh13	C5ar1		Chd4	Slc12A2	Msx2
Hipk3	Ddit4	Cadm1		Clca2	Tmprss2	Pfkfb3
Hspa1b	Krt8	Cd44		Cldn8	Galnt2	Slpi
Ide	Lasp1	Cd53		Col9A1	Nnat	Stat5A
Il13ra1	Nr1d2	Cdt1		Comt1	Npr3	Stc2
Krt19	Shc1	Cel		Csn1S1	Nr2C2	Wfdc2
Ltf	Slc12a2	Chd4		Csnk2A1	Padi2	Adam8
Ly6d	Stc2	Ctss		Cxcl15	Pcyt1A	Asf1B
Padi2	Tbx3	Cxcr4		Ddx6	Pof1B	Cd44
Papss2	Tgm2	Cybb		Dnmt3A	Prom1	Chek1
Pde4dip	Adamts4	Fbn2		Enah	Prom2	Cxcr4
Pfkfb3	Atf3	Fyb		F3	Ptpn5	Dhcr7
Rarg	Ccl2	Galnt2		Fxyd2	Ripk4	Ect2
Rhpn2	Ccl5	Galr1		Gata3	Rnd3	Eif2C2
Rnd3	Ccnd2	Gp49a///Lilrb4		Gipc2	Rnf128	Gzmb
Rnf141	Lims1	Gzma		Gzma	Rsad2	Idi1
Slpi	Mmp12	Il7r		H19	S100A8	Itgb2
Socs2	Pafah1b1	Iqgap2		Hspa1B	Scd2	Kif11
Tmed3	Prg4	Itgb2		Il13Ra1	Serpinb11	Lcp1
Tsc22d1	Tlr1	Kif11		Il33	Slc2A5	Lst1
Usp9x	Vcan	Lcp2		Isyna1	Slpi	Mcm6
Wnt4	Akr1c14	Lipg		Itgav	Sncg	Ms4A7
Cd48	Areg	Ly86		Kcnb1	Socs2	Nuf2
Chek1	Cldn8	Mbp		Kcnk1	Spon1	Ptprc
Eif2c2	Fbxo21	Mfap4		Kcnk3	Spp1	Rad51
Glipr1	Gata3	Mvd		Krt19	Stc2	Rrm2
Gpnmb	Hif1a	Ncf2		Krt7	Tgm2	S100A8
Gzmb	Itgav	Ncf4		Krt8	Tmed3	Slc40A1
Idi1	Krt7	Pim1		Ktn1	Tmem30B	Spp1
Laptm5	Mme	Plau		Lasp1	Tmem56	Stmn1
Lcp1	Osmr	Ptger3		Lcp1	Tmem79	Top2A
Lgals3	Runx1	Ptges		Lgals12	Tnfaip6	Uhrf1
Lpxn	Slc2a3	Ptprc		Lgals3	Vcan	
Malat1	Slc7a2	Rap2b		Lipg	Wfdc2	
Mapk1	Spon1	Reln		Ltf	Zbtb8A	
Mcm5	Stat5a	Rrm2		Ly6D		

* Genes listed in alphabetical order.

[a] TGF**β** signaling and interaction database (http://actin.ucd.ie/tgfbeta/) and [20]

[b] McBryan et al. 2007 [19]

[c] Abba et al. 2010 [18]
