# Supplementary material for: Genetic Differences in Transcript Responses to Low-Dose Ionizing Radiation Identify Tissue Functions Associated with Breast Cancer Susceptibility
Source: PLoS One. 2012 Oct 15;7(10):e45394. doi: 10.1371/journal.pone.0045394 (PMC3471924; doi:10.1371/journal.pone.0045394)
Supplement: Table S7 — Summary of quantitative RT PCR confirmations of microarray findings. (PDF) [file pone.0045394.s011.pdf]

Table S7. Summary of quantitative RT PCR confirmation of microarray findings.

**Baseline expression differences in blood of BALB/c vs C57BL/6 mice**		Fold changes listed in $\log_2$ relative average expression in four C57BL/6 mice				
**Gene**	**Function**	**Taqman AOD ID**	**BALB/c LD #1**	**BALB/c LD #2**	**BALB/c LD #3**	**BALB/c LD #4**
PARP3	DNA stability	Mm00467486_m1	-1.27	-1.17	-0.83	-1.12
PARP3	DNA stability	Mm01232604_m1	-1.80	-1.00	-1.14	-1.57
MSH5	DNA stability	Mm00488974_m1	2.34	2.65	2.21	2.34
SMC6	DNA stability	Mm01273370_m1	1.59	1.80	1.74	2.32

Early (4 hr) Radiation Response		Fold changes listed in log_2 relative to Sham						
**Gene**	**Function**	**Taqman AOD ID**	**BALB/c LD #1***	**BALB/c LD #2**	**BALB/c LD #3**	**BALB/c LD #4**	**BALB/c LD #5**	**BALB/c LD #6***
CD24	Mammary dev**	Mm00782538_sH	0.91	1.29	2.03	1.14	1.27	0.98
KRT19	Mammary dev	Mm00492980_m1	0.44	1.02	0.93	1.43	1.26	0.63
WNT4	Mammary dev	Mm01194003_m1	1.07	1.20	0.78	0.84	1.34	0.38
IDO1	Mammary dev	Mm00492586_m1	2.66	-0.84	2.41	4.30	-0.28	4.04
AREG	Mammary dev	Mm01354339_m1	-1.30	1.84	1.07	2.43	2.40	2.03
CDT1	Mitosis	Mm00466006_m1	-0.86	-1.14	-0.72	-1.66	-0.27	-1.27
HIF1a	Hypoxia	Mm00468878_m1	0.52	0.83	0.77	0.97	1.25	0.57
STC2	Hypoxia	Mm00441561_m1	0.35	1.18	1.03	1.11	0.74	1.72
RAB20	Hypoxia	Mm01216807_m1	0.62	0.91	0.27	0.74	0.33	1.26
FXYD2	Ion transport regulator	Mm00446358_m1	1.59	1.60	1.35	2.06	1.24	1.75
IRF8	Immune Response	Mm00492567_m1	-0.10	-0.51	-0.65	-1.39	-0.84	-0.88
IL7R	Immune Response	Mm00434295_m1	-0.66	-1.38	-1.12	-1.26	-1.25	-1.45
TREM2	Immune Response	Mm00451744_m1	1.04	-1.05	-1.00	-0.43	-0.27	-0.54

*Animals highlighted red were not analyzed on microarray; LD = Low Dose animals
** Mammary gland development

**Early (4 hr) Radiation Response**		Fold changes listed in log_2 relative to Sham						
**Gene**	**Function**	**Taqman AOD ID**	**BALB/c HD #1***	**BALB/c HD #2**	**BALB/c HD #3**	**BALB/c HD #4**	**BALB/c HD #5**	**BALB/c HD #6***
CD24	Mammary dev**	Mm00782538_sH	-0.20	-1.35	-1.40	-2.52	-1.79	-0.65
KRT19	Mammary dev	Mm00492980_m1	-1.42	-1.53	-0.78	-2.70	-1.75	-1.10
WNT4	Mammary dev	Mm01194003_m1	-0.61	-1.20	0.50	-2.72	-1.56	-0.96
IDO1	Mammary dev	Mm00492586_m1	-0.91	-0.06	0.37	-0.69	-0.91	-0.52
AREG	Mammary dev	Mm01354339_m1	-2.21	-2.78	-1.64	-5.03	-3.45	-2.73
CDT1	Mitosis	Mm00466006_m1	-1.16	-1.81	-0.98	-1.57	-1.63	-0.76

* Animals highlighted red were not analyzed on microarray; HD = High Dose animals
** Mammary gland development

**1-Month Radiation Response**		Fold changes listed in log_2 relative to Sham								
			**C57BL/6 LD #1**	**C57BL/6 LD #2**	**C57BL/6 LD #3**	**C57BL/6 LD #4**	**C57BL/6 HD #1**	**C57BL/6 HD #2**	**C57BL/6 HD #3**	**C57BL/6 HD #4**
SPRR1A	Regeneration	Mm01962902_s1	6.34	7.54	4.27	6.54	3.34	1.88	5.10	2.07
CDKN1A	Mitosis	Mm00432448_m1	0.98	1.04	0.21	0.64				
SOX9	Mitosis	Mm00448840_m1	-0.79	-0.80	-0.43	0.26				
SKP2	Mitosis	Mm00449925_m1	-0.59	-0.45	-0.53	-0.34				
CCNA2	Mitosis	Mm00438064_m1	-1.56	-1.60	-1.67	-0.42				

			**BALB/c LD #1**	**BALB/c LD #2**	**BALB/c LD #3**	**BALB/c LD #4**
CDKN1A	Mitosis	Mm00432448_m1	-0.129	-0.5425	-0.168	-0.642
UCP1	Thermogenesis	Mm01244860_m1	1.07	1.49	0.62	3.05
