# Supplementary material for: Genetic Differences in Transcript Responses to Low-Dose Ionizing Radiation Identify Tissue Functions Associated with Breast Cancer Susceptibility
Source: PLoS One. 2012 Oct 15;7(10):e45394. doi: 10.1371/journal.pone.0045394 (PMC3471924; doi:10.1371/journal.pone.0045394)
Supplement: Text S1 — Supplementary methods. (PDF) [file pone.0045394.s012.pdf]

Genetic differences in transcript responses to low-dose ionizing radiation identify molecular functions associated with breast cancer susceptibility

Antoine M. Snijders, Francesco Marchetti, Sandhya Bhatnagar, Nadire Duru, Ju Han, Zhi Hu, Jian-Hua Mao, Joe Gray and Andrew J. Wyrobek

Text S1. Methods.

RNA Isolations and microarray hybridization

Blood and mammary gland tissues were collected from 6 animals per dose group for each strain. Blood was collected from the heart into a heparinized syringe and immediately transferred to RNA later (Ambion). RNA was isolated using the Blood- Ribopure blood kit (Ambion) followed by Globin clear kit following the manufacturer's recommendations. . The 4th mammary gland pair was harvested and the inguinal lymph node as well as a second lymph node often present in the distal part of the inguinal mammary gland were removed Mammary tissues were snap frozen in liquid nitrogen within 10 minutes of the euthanasia procedure. Total RNA was isolated by homogenizing the frozen tissue in Trizol reagent (Invitrogen) followed by phase separation using chloroform. RNA was further purified by Qiagen's RNeasy mini kit (74104) and DNA was removed using Qiagen's DNase free kit (79254). RNA samples with RIN numbers greater than 7 were used for further analyses. Microarray hybridizations were performed on four mice for each dose group in the Lawrence Berkeley National Laboratory's HTA facility using Affymetrix's HT Mouse Genome 430A 96-Array Plate. Robust multi-array average (RMA) was then used to create an expression matrix from Affymetrix data. The normalized unscaled standard error (NUSE) plot was generated to visualize the chip-wise distribution of standard error estimates obtained for each gene on each array when performing the robust multichip probe-level fit. Any array with the upper quartile NUSE

value greater than 1.10 was removed from the analysis. A statistical hypothesis testing method based on moderated t-statistic is used for detecting differential expression, which is implemented through the R *limma* package. Gene fold-changes were calculated with respect to sham irradiated animals and gene lists were prepared based on fold-change (log2 0.58) and p-value (0.1 for low-dose; 0.01 for high-dose). The p-value of approximately 80% of the low-dose responsive genes was ≤ 0.05. We choose to use the less-conservative p-value of 0.1 to allow for sufficient numbers of genes to be included in downstream bioinformatics analyses, which used more stringent p-values. Gene lists were analyzed using Ingenuity Pathway Analysis, the L2L microarray comparison tool (http://depts.washington.edu/l2l/), KEGG pathway analysis (http://bioinfo.vanderbilt.edu/webgestalt/) and DAVID GO gene ontology (http://david.abcc.ncifcrf.gov/; $p \leq 0.05$) [1]. Select expression array findings were confirmed using quantitative RT PCR analysis following standard methods. β−Actin expression was used as endogenous control. Fold changes were calculated with respect to average of four sham replicates and represented in log2.

Mouse and human database comparisons

Low dose BALB/c and C57BL/6 gene lists were compared against a gene expression signature containing pubertal mammary gland development genes [2], known TGFβ-responsive genes (http://actin.ucd.ie/tgfbeta/ and [3]), and a meta-analysis of 42 gene expression signatures of breast cancer [4].NextBio (http://www.nextbio.com/) was used to retrieve the direction of expression of low-dose genes in DCIS and breast cancer [5-9]. Genes were queried in NextBio applying the following filters: “human: as organism,

“breast cancer” as key word and “disease versus non-disease” option to find the directionality of expression in transcript profiling studies of cancer versus non-cancer breast tissues.

Baseline strain difference signature and low-dose expression signature at 1-month after irradiation as prognostic indicators in breast cancer patients.

Human homologs of mouse genes were identified for two separate gene lists: (1) baseline gene expression differences between BALB/c and C57BL/6 in mammary gland and blood (131 genes) and (2) up-regulated late BALB/c genes that were not up-regulated in C57BL/6 (105 genes). The baseline and low-dose gene lists were compared against U133A Affymetrix expression array, which identified 94 and 96 common genes, respectively. Expression levels of these genes in human breast cancer patients of a curated breast cancer data set (GSE1456) were summed and mean-normalized [10]. The median expression value was used as a cut-point to assign patients to either “above-median” expression and “below-median” expression and to assess patient outcome, significance was tested using chi-square test. The average of the mean-normalized summed expression values was calculated for each breast cancer subtype. Kaplan-Meier disease-free survival curves were generated comparing the above-median patient group with the below-median patient group in two curated breast cancer data sets (GSE1456 alone and combined with GSE6532) [10, 11]. Log-rank tests were performed to compare the difference in disease-free survival between patients in the two clusters.

Immunohistochemical analyses

Following heat-mediated antigen retrieval, sections were processed for immunohistochemistry by blocking with serum (1:10 dilution) corresponding to the species of the biotinylated secondary antibody. Sections were incubated overnight with SOX9 primary antibody at 1:200 dilution (Millipore, AB5535) or F4/80 primary antibody at 1:500 dilution (Abcam, Ab6640). Staining was visualized using the Vectastain ABC kit (Vector Labs) and DAB/H_2O_2. Sections were counterstained with hematoxylin, rinsed in deionized water, differentiated in a 1% acid alcohol solution, rinsed in deionized water and blued in Scott's water. Sections were rinsed in deionized water, dehydrated through graded alcohols and cleared in xylene. Sections were coverlipped using Permount. For each mammary gland, approximately 1000 luminal and myoepithelial cells were counted from 2 mice per group, blinded.

Design limitations

Our study was limited in that we used only two strains that differed in their radiation sensitivity, and it remains to be investigated whether similar damage responses will be identified in mammary tissue of other mammary cancer sensitive and resistant strains of mice (e.g., C3H and SPRET/EI). The time-points chosen for our studies (4 hrs and 1 month after the last irradiation) are far removed from the time when low-dose radiation induced mammary cancers manifest themselves, and a more detailed post radiation time response study is warranted. Our transcript profiles used a gross mixture of mammary cell types, which limits our ability to assign functions to specific cell types. To reduce mammary tissue complexity, we removed the inguinal lymph node before transcriptional analyses.

References

1. Dennis, G, Jr., et al., DAVID: Database for Annotation, Visualization, and Integrated Discovery. Genome Biol, 2003. **4**(5): p. P3.
2. McBryan, J, et al., ERalpha-CITED1 co-regulated genes expressed during pubertal mammary gland development: implications for breast cancer prognosis. Oncogene, 2007. **26**(44): p. 6406-19.
3. Xu, XL and AM Kapoun, Heterogeneous activation of the TGFbeta pathway in glioblastomas identified by gene expression-based classification using TGFbeta-responsive genes. J Transl Med, 2009. **7**: p. 12.
4. Abba, MC, E Lacunza, M Butti, and CM Aldaz, Breast cancer biomarker discovery in the functional genomic age: a systematic review of 42 gene expression signatures. Biomark Insights, 2010. **5**: p. 103-18.
5. Cheng, AS, et al., Epithelial progeny of estrogen-exposed breast progenitor cells display a cancer-like methylome. Cancer Res, 2008. **68**(6): p. 1786-96.
6. Emery, LA, et al., Early dysregulation of cell adhesion and extracellular matrix pathways in breast cancer progression. Am J Pathol, 2009. **175**(3): p. 1292-302.
7. Hu, Z, et al., A compact VEGF signature associated with distant metastases and poor outcomes. BMC Med, 2009. **7**: p. 9.
8. Pedraza, V, et al., Gene expression signatures in breast cancer distinguish phenotype characteristics, histologic subtypes, and tumor invasiveness. Cancer. **116**(2): p. 486-96.
9. Yu, K, et al., A precisely regulated gene expression cassette potently modulates metastasis and survival in multiple solid cancers. PLoS Genet, 2008. **4**(7): p. e1000129.
10. Pawitan, Y, et al., Gene expression profiling spares early breast cancer patients from adjuvant therapy: derived and validated in two population-based cohorts. Breast Cancer Res, 2005. **7**(6): p. R953-64.
11. Loi, S, et al., Definition of clinically distinct molecular subtypes in estrogen receptor-positive breast carcinomas through genomic grade. J Clin Oncol, 2007. **25**(10): p. 1239-46.
